# Intramural Healthcare Consumption and Costs After Traumatic Brain Injury: A Collaborative European NeuroTrauma Effectiveness Research in Traumatic Brain Injury (CENTER-TBI) Study

**DOI:** 10.1089/neu.2022.0429

**Published:** 2023-09-29

**Authors:** Z.L. Rana Kaplan, Marjolein van der Vlegel, Jeroen T.J.M. van Dijck, Dana Pisică, Nikki van Leeuwen, Hester F. Lingsma, Ewout W. Steyerberg, Juanita A. Haagsma, Marek Majdan, Suzanne Polinder

**Affiliations:** ^1^ Department of Public Health, Erasmus University Medical Center, Rotterdam, The Netherlands.; ^2^ Department of Neurosurgery, University Neurosurgical Center Holland (UNCH), Leiden University Medical Center & Haaglanden Medical Center & HAGA Teaching Hospital, Leiden/The Hague, The Netherlands.; ^3^ Department of Biomedical Data Sciences, Leiden University Medical Center, Leiden, The Netherlands.; ^4^ Institute for Global Health and Epidemiology, Department of Public Health, Trnava University, Trnava, Slovakia.

**Keywords:** healthcare consumption, healthcare costs, hospital costs, traumatic brain injury;

## Abstract

Traumatic brain injury (TBI) is a global public health problem and a leading cause of mortality, morbidity, and disability. The increasing incidence combined with the heterogeneity and complexity of TBI will inevitably place a substantial burden on health systems. These findings emphasize the importance of obtaining accurate and timely insights into healthcare consumption and costs on a multi-national scale. This study aimed to describe intramural healthcare consumption and costs across the full spectrum of TBI in Europe. The Collaborative European NeuroTrauma Effectiveness Research in Traumatic Brain Injury (CENTER-TBI) core study is a prospective observational study conducted in 18 countries across Europe and in Israel. The baseline Glasgow Coma Scale (GCS) was used to differentiate patients by brain injury severity in mild (GCS 13–15), moderate (GCS 9–12), or severe (GCS ≤8) TBI. We analyzed seven main cost categories: pre-hospital care, hospital admission, surgical interventions, imaging, laboratory, blood products, and rehabilitation. Costs were estimated based on Dutch reference prices and converted to country-specific unit prices using gross domestic product (GDP)-purchasing power parity (PPP) adjustment. Mixed linear regression was used to identify between-country differences in length of stay (LOS), as a parameter of healthcare consumption. Mixed generalized linear models with gamma distribution and log link function quantified associations of patient characteristics with higher total costs. We included 4349 patients, of whom 2854 (66%) had mild, 371 (9%) had moderate, and 962 (22%) had severe TBI. Hospitalization accounted for the largest part of the intramural consumption and costs (60%). In the total study population, the mean LOS was 5.1 days at the intensive care unit (ICU) and 6.3 days at the ward. For mild, moderate, and severe TBI, mean LOS was, respectively, 1.8, 8.9, and 13.5 days at the ICU and 4.5, 10.1, and 10.3 days at the ward. Other large contributors to the total costs were rehabilitation (19%) and intracranial surgeries (8%). Total costs increased with higher age and greater trauma severity (mild; €3,800 [IQR €1,400–14,000], moderate; €37,800 [IQR €14,900–€74,200], severe; €60,400 [IQR €24,400–€112,700]). The adjusted analysis showed that female patients had lower costs than male patients (odds ratio (OR) 0.80 [CI 0.75–1.85]). Increasing TBI severity was associated with higher costs, OR 1.46 (confidence interval [CI] 1.31–1.63) and OR 1.67 [CI 1.52–1.84] for moderate and severe patients, respectively. A worse pre-morbid overall health state, increasing age and more severe systemic trauma, expressed in the Injury Severity Score (ISS), were also significantly associated with higher costs. Intramural costs of TBI are significant and are profoundly driven by hospitalization. Costs increased with trauma severity and age, and male patients incurred higher costs. Reducing LOS could be targeted with advanced care planning, in order to provide cost-effective care.

## Introduction

Each year, ∼1,500,000 people with traumatic brain injury (TBI) are hospitalized in the European Union, and ∼57 000 die as a result of a TBI, translating on average into 287 hospital admissions and ∼12 deaths per 100 000 inhabitants.^1,2^ The population-based incidence that includes those injuries that are not treated at hospitals can even be as high as 790 per 100,000.^3^ The incidence of TBI may further increase in the future, mainly driven by an increasing incidence of falls within the growing elderly population in most high-income countries, and the increasing number of road traffic incidents in low-to-middle-income countries where the implementation and effectiveness of preventative measures are outpaced by the rapid increase in motorization.^4–7^ The increasing number of cases combined with the heterogeneity and complexity of TBI will inevitably put a substantial burden on health systems, as the consumption of specialized acute care and long-term rehabilitation or chronic care will concomitantly increase.^1,8^

The healthcare costs of TBI, driven by cost prices and the healthcare consumption of patients, will cause major economic and societal challenges, as estimates indicate the worldwide annual economic burden of TBI to be US $400 billion dollars, which is ∼0.5% of the gross world product.^1,9–11^ This is of concern, as the associated increase of costs occurs at a time when there is a global shortage in healthcare personnel, healthcare spending budgets are under pressure, and justification of healthcare expenses will become increasingly important.^12–15^ It is therefore essential to obtain accurate and timely insight into healthcare consumption after TBI, and the cost effectiveness of TBI treatments, to optimize future allocation of restricted healthcare budgets.^16^ In view of these trends, cost studies have gained more importance, as measurement of healthcare consumption and accompanied costs serves as a fundament for improvement of access to and delivery of healthcare and for identification of potential savings.^1,2,8,17^

Published studies report in-hospital costs of patients with TBI to range from $3,079 to $7,800 (€2,721–6,893) for mild TBI patients^16,18–21^ and from $2,130 to $401,808 (€1,882–355,117) for severe TBI patients.^17^ Hospital costs increase with higher TBI severity and are mostly driven by the length of stay at the hospital.^16–21^ Unfortunately, the interpretation, comparability, and generalizability of these study results are difficult and limited. Most available research on costs after TBI frequently suffers from major methodological heterogeneity and inadequate quality, and is commonly restricted to one TBI severity level. Additionally, implementation of clinical guideline recommendations and personnel costs differ across hospitals and countries, resulting in different treatment practices and cost patterns.^9,10,16,22^ As measurement of healthcare consumption and costs after TBI differs among countries, researchers usually assess strictly local or national expenses, which limits the understanding and possibility of comparisons on a multi-national scale. In order to address these shortcomings, this study aimed to provide a detailed overview of intramural healthcare consumption and healthcare costs arising from hospital admission and inpatient rehabilitation, across the full spectrum of TBI in Europe.

## Methods

### Study design and patients

The Collaborative European NeuroTrauma Effectiveness Research in Traumatic Brain Injury (CENTER-TBI) core study is a prospective longitudinal non-randomized observational study, registered at clinicaltrials.gov NCT02210221, which included patients with TBI from 18 countries across Europe and in Israel between 2014 and 2017. Inclusion criteria were: (1) a clinical diagnosis of TBI, (2) a clinical indication for a computed tomographic (CT) scan, (3) presentation within 24 h of injury, and (4) informed consent obtained according to local and national policies. Patients were excluded if they had a severe pre-existing neurological disorder that would confound outcome assessments. For this particular study, patients from Israel and those <16 years of age were excluded. Ethical approval for the CENTER-TBI study was obtained from all responsible medical ethical committees, and informed consent procedures followed applicable regulations.^23^

### Clinical data

Clinical data were prospectively collected by local research staff using electronic case report forms (eCRF). Data were de-identified using a randomly generated Global Unique Patient Identifier (GUPI) and stored on a secured database by the International Neuroinformatics Coordinating Facility (INCF) (www.incf.org) in Stockholm, Sweden. Data were extracted in January 2021 (version 3.1) and included demographic characteristics, trauma and injury information, results of neurological assessment, imaging, and patient outcomes. Using the baseline Glasgow Coma Scale (GCS) score, patients were classified into three categories of TBI severity: GCS 13–15 (mild TBI), GCS 9–12 (moderate TBI), and GCS 3–8 (severe TBI).^24^ The baseline GCS score is a derived variable and represents the total GCS score for baseline risk adjustment. The systemic injury severity score (ISS) was categorized into three groups: ISS ≤16 (minor injury), ISS 17–25 (major injury), and ISS >25 (critical injury).^25^ Pre-injury health status was classified using the American Society of Anesthesiologists (ASA) physical status classification.^26^ Brain injury is further described according to the Abbreviated Injury Scale (AIS) and classified as minor, moderate, serious, severe, critical, or unsurvivable.^27^

### Healthcare consumption

Healthcare consumption data were extracted following the same procedure as with clinical data. The healthcare consumption of patients included seven main healthcare service categories: (1) pre-hospital care, including ambulance transportation and, for secondary referral patients, costs of TBI-related admission and any emergent surgical interventions in the “referring hospital,” before admission to a CENTER-TBI study hospital; (2) hospital admission, including initial assessment and care at the emergency room (ER) and length of stay (LOS) in days at the ward or ICU; (3) all surgical interventions, both intra- and extra-cranial; (4) imaging of the brain; (5) laboratory; (6) blood products; and (7) rehabilitation; including only LOS at an inpatient rehabilitation center. Healthcare consumption of outpatient rehabilitation care facilities was not included. The transitions of care forms, in which the care pathway of patients was registered, were used to extract the in-hospital LOS of patients. Inpatient rehabilitation LOS was extracted using the transitions of care forms and patient-reported outcome forms. Missing LOS at the ward, ICU, and rehabilitation were imputed using single imputation. All healthcare services registered within CENTER-TBI and included in this study are reported in Supplementary Table S1.

### Healthcare costs

Because of the unavailability of country-specific unit prices for each healthcare service, Dutch reference prices were used as fundament for this study. In addition, definitions, calculations, and sources of country-specific unit prices may vary (e.g., unit prices can differ based on the inclusion/exclusion of personnel costs), which could potentially lead to an over- or underestimation of costs when such unit prices are used. For example, it was found that the reported monthly salary for a senior resident ranged from a low between €500 and €800 in Eastern Europe to a high of €7900 in Norway.^28^ By using a uniform price list, this study focuses on differences in healthcare consumption rather than price differences among countries.

Reference prices were extracted from the Dutch Guidelines for economic healthcare evaluations.^29^ Reference prices not mentioned in the Dutch Guidelines were complemented using unit prices reported by the Netherlands Healthcare Authority or by using the average national price, based on declared fees^30,31^ (Supplementary Table S1). First, using the Dutch national general consumer price index, all reference prices were corrected to EURO 2017, the last year of patient inclusion (Supplementary Table S2).^32^ Second, in order to calculate the economic burden of a patient with TBI within Europe, the Dutch reference prices were converted to country-specific unit prices by correcting the Dutch reference prices for the purchasing power parity (PPP) for the general domestic product (GDP) (Supplementary Table S3). The GDP-PPP is the standard measure when comparing differences in life standards among countries.^33^

Third, the total intramural healthcare costs were calculated by multiplying the number of healthcare units (e.g. length of days at ward and ICU for hospitalization costs) with the corresponding reference price, according to country of admission. See Supplemental Methods, Supplementary Tables S2 and S3 for further details about the calculations.

### Statistical analysis

Data were analyzed using descriptive statistics. Baseline characteristics of patients are based on crude data and presented as absolute numbers and percentages. Continuous variables are presented as medians (interquartile range [IQR]) and means (standard deviation [SD]). Median and mean prices were rounded to hundreds. To compare continuous and categorical variables across all subgroups, the Kruskal–Wallis test and the χ^2^ test were applied respectively. A *p* value <0.05 was considered statistically significant. Healthcare consumption (i.e., LOS at ICU, ward, and rehabilitation unit) and total healthcare costs were presented for the total study population, including all severities, and according to TBI severity.

Missing data were statistically imputed based on correlations among baseline characteristics, healthcare consumption, in-hospital mortality, and Glasgow Outcome Scale Extended (GOSE) score at 6 months using the mice package in R.^34^ To determine between-country differences in ICU and ward LOS, a mixed linear regression model was applied, with results presented in forest plots. The country effect was included in the model as a random intercept, and case-mix adjustment was performed using variables in the International Mission for Prognosis and Analysis of Clinical Trials in TBI (IMPACT) prognostic model: age, pupils, GCS score, hypoxia, hypotension, traumatic subarachnoid hemorrhage, epidural hemorrhage, Marshall CT classification, hemoglobin, and glucose measurements.^35^ Countries including fewer than five patients per severity group were excluded from this analysis.

We used a mixed general linear model (GLM) with gamma distribution and log link function to determine which baseline characteristics were associated with the total intramural healthcare costs. GLM models are recommended for use in linear regression of costs data, as they provide parametric methods of analysis in which non-normal distributions can be specified.^36^ A random effect for country was added to both the univariable and multi-variable models to account for between-country differences in costs. Statistical analysis were performed in STATA and R version 4.0.4.^37,38^

## Results

### Patient population

After exclusion of patients from Israel and those <16 years of age, a total of 4349 out of 4509 CENTER-TBI patients were included in this study. Patients were mostly male (67%), with a median age of 51 years (IQR 32–67). Of the total population, 27% were ≥65 years of age (Table 1). A total of 457 patients (11%), had severe systemic disease, of whom 291 (64%), were ≥65 years of age. The most common causes of TBI were falls (45%), road traffic incidents (37%), and violence (6%). Of the 4349 patients, 2854 (66%) had mild TBI, 371 (9%) had moderate TBI, and 962 (22%) had severe TBI. Pupillary reaction was abnormal in 10% of patients. Intracranial CT abnormalities were found in 55%, with traumatic subarachnoid hemorrhage (41%), contusions (31%) and acute subdural hematoma (26%) as the most common abnormalities. Total in-hospital mortality was 7%, increasing from 1% for patients with mild TBI, to 22% for those with severe TBI.

**Table 1.** Baseline Characteristics of Patients According to Trauma Severity

Patient characteristics	Trauma severity								*p* value
	Mild		Moderate		Severe		Total		
	No.	(%)	No.	(%)	No.	(%)	No.	(%)	
Total	2854	65.6%	371	8.5%	962	22.1%	4349	100.0%	
Sex									<0.001
Male	1835	64.3%	254	68.5%	726	75.5%	2926	67.3%	
Female	1019	35.7%	117	31.5%	236	24.5%	1423	32.7%	
Age									<0.001
Median [IQR], years	53 [33-68]		55 [35-70]		47 [29-64]		51 [32-67]		
16-25 years	449	15.7%	52	14.0%	197	20.5%	725	16.7%	
26-40 years	501	17.6%	64	17.3%	190	19.8%	783	18.0%	
41-64 years	1087	38.1%	132	35.6%	358	37.2%	1648	37.9%	
≥65 years	817	28.6%	123	33.2%	217	22.6%	1193	27.4%	
Medical history									<0.001
Healthy patient	1563	54.8%	181	48.8%	528	54.9%	2352	54.1%	
Mild systemic disease	951	33.3%	130	35.0%	275	28.6%	1401	32.2%	
Severe systemic disease	310	10.9%	47	12.7%	97	10.1%	460	10.6%	
*Missing*	30	1.1%	13	3.5%	62	6.4%	136	3.1%	
Cause of injury									<0.001
Road traffic accident	973	34.1%	139	37.5%	456	47.4%	1619	37.2%	
Fall	1392	48.8%	157	42.3%	352	36.6%	1955	45.0%	
Violence	186	6.5%	22	5.9%	28	2.9%	244	5.6%	
Self-harm	15	0.5%	6	1.6%	23	2.4%	48	1.1%	
Other	240	8.4%	36	9.7%	66	6.9%	362	8.3%	
*Missing*	48	1.7%	11	3.0%	37	3.8%	121	2.8%	
Brain AIS									<0.001
Minor	773	27.1%	14	3.8%	8	0.8%	803	18.5%	
Moderate	470	16.5%	8	2.2%	18	1.9%	503	11.6%	
Serious	1081	37.9%	42	11.3%	29	3.0%	1183	27.2%	
Severe	371	13.0%	131	35.3%	179	18.6%	714	16.4%	
Critical	130	4.6%	166	44.7%	653	67.9%	1000	23.0%	
Unsurvivable	2	0.1%	5	1.3%	70	7.3%	86	2.0%	
*Missing*	27	0.9%	5	1.3%	5	0.5%	60	1.4%	
ISS									<0.001
Minor (≤16)	1973	69.1%	169	45.6%	667	69.3%	1256	28.9%	
Major (17-25)	506	17.7%	100	27.0%	223	23.2%	862	19.8%	
Critically injured (>25)	351	12.3%	97	26.1%	67	7.0%	2167	49.8%	
*Missing*	24	0.8%	5	1.3%	5	0.5%	64	1.5%	
Baseline pupillary reaction									<0.001
Both reacting	2655	93.0%	315	84.9%	618	64.2%	3654	84.0%	
One reacting	46	1.6%	15	4.0%	95	9.9%	162	3.7%	
Non-reacting	28	1.0%	20	5.4%	216	22.5%	277	6.4%	
*Missing*	125	4.4%	21	5.7%	33	3.4%	256	5.9%	
CT abnormalities									
Any CT abnormality									<0.001
Absent	1443	50.6%	31	8.4%	59	6.1%	1575	36.2%	
Present	1217	42.6%	287	77.4%	789	82.0%	2388	54.9%	
Cisternal compression	124	4.3%	89	24.0%	380	39.5%	627	14.4%	<0.001
Midline shift	103	3.6%	77	20.8%	252	26.2%	455	10.5%	<0.001
Subarachnoid hemorrhage	808	28.3%	244	65.8%	663	68.9%	1793	41.2%	<0.001
Epidural hematoma	207	7.3%	73	19.7%	128	13.3%	425	9.8%	<0.001
Acute subdural hematoma	472	16.5%	166	44.7%	442	45.9%	1126	25.9%	<0.001
Diffuse axonal injury	166	5.8%	48	12.9%	212	22.0%	443	10.2%	<0.001
Contusion	563	19.7%	207	55.8%	502	52.2%	1336	30.7%	<0.001
*No CT scan performed*	129	4.5%	35	9.4%	80	8.3%	261	6.0%	
In-hospital mortality									<0.001
No	2034	71.3%	327	88.1%	742	77.1%	3216	73.9%	
Yes	35	1.2%	40	10.8%	207	21.5%	310	7.1%	
*Missing*	785	27.5%	4	1.1%	13	1.4%	823	18.9%	
GOSE-6 months disability									<0.001
1	89	3.1%	74	19.9%	273	28.4%	470	10.8%	
2-3	97	3.4%	33	8.9%	171	17.8%	314	7.2%	
4	83	2.9%	25	6.7%	57	5.9%	174	4.0%	
5	169	5.9%	47	12.7%	110	11.4%	339	7.8%	
6	244	8.5%	36	9.7%	90	9.4%	383	8.8%	
7	528	18.5%	39	10.5%	78	8.1%	658	15.1%	
8	1160	40.6%	63	17.0%	66	6.9%	1325	30.5%	
* Missing*	484	17.0%	54	14.6%	117	12.2%	686	15.8%	

A total of 157 patients were missing information on the baseline Glasgow Coma Scale score.

The *p* value assesses the null hypothesis of no differences among the mild, moderate, and severe subgroups.

IQR, interquartile range; AIS, Abbreviated Injury Score; ISS, Injury Severity Score; CT, computed tomography; GOSE, Glasgow Outcome Scale Extended.

### Healthcare consumption

**FIG. 1.**
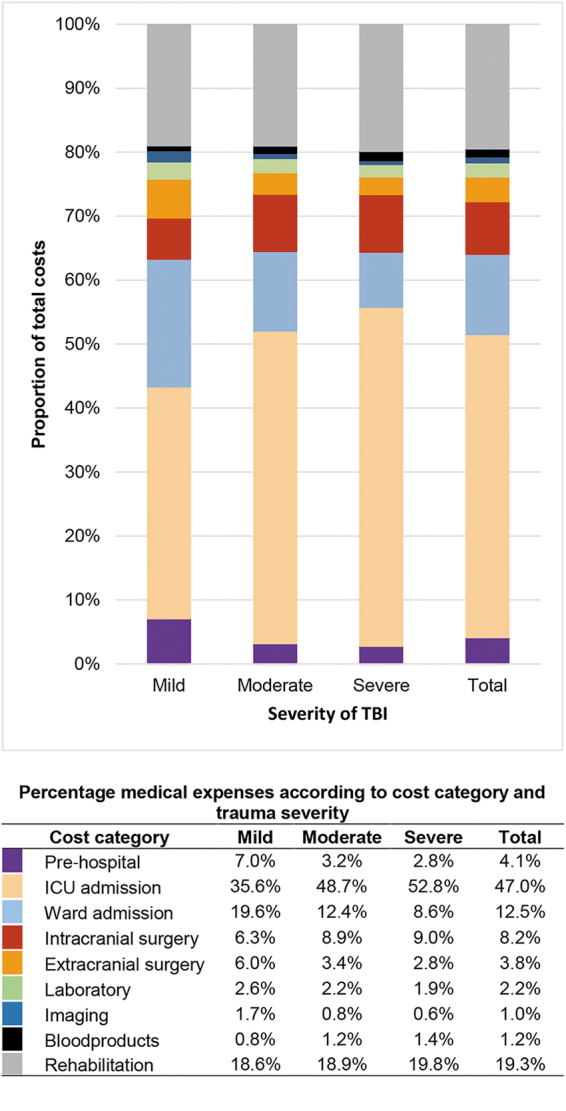
Proportion of mean total intramural costs per cost- category according to severity of traumatic brain injury (TBI). The proportion of the total intramural costs from each cost category are plotted in a histogram for each TBI severity level separately. The exact percentage for each cost category (including pre-hospital costs, intensive care unit and ward admission costs, intra- and extracranial surgery costs, laboratory costs, imaging costs, blood products costs, and rehabilitation costs) are presented in the table below the figure. For example, of the total costs within the mild TBI category, 7% of the expenses were from pre-hospital costs.

Hospital admission (i.e. including ICU and ward admission) accounted for over half (60%) of the mean total intramural costs (mild TBI: €8,200 [55%], moderate TBI: €33,400 [61%], severe TBI: €48,500 [61%]), of which 47% were related to ICU admission and 13% were related to ward admission (Fig. 1 and Supplementary Table S4). For the total study population, the mean LOS at the ICU and ward were 5.1 and 6.3 days respectively (Table 2). For mild, moderate, and severe TBI, mean LOS was 1.8, 8.9, and 13.5 days in the ICU and 4.5, 10.1, and 10.3 days on the ward, respectively. The mean LOS for inpatient rehabilitation was 13.5 days for the total population and 5.8, 22.1, and 32.6 days, respectively, for mild, moderate, and severe TBI. Rehabilitation costs (19%; €6,400) and intracranial surgeries (8%; €2,700) were also large cost contributors (Fig. 1 and Supplementary Table S4). Costs for all categories were higher for each TBI severity level. Proportion of total costs related to ICU admission and intracranial surgery increased with TBI severity, while proportion of costs related to ward admission, pre-hospital expenses, and extracranial surgery decreased. Patients who sustained TBI as a result of self-harm had the longest ICU and ward LOS (11 and 17 days, respectively). Patients who died during admission had higher median total costs (€18.900 vs. €8,500) (Table 2).

**Table 2.** Median Intramural Costs for Each Cost Category According to Baseline Characteristics

Patient characteristics	Cost category																																
	Total costs			Pre-hospital			Intracranial			Extracranial			Hospitalization			ICU (days)		Ward (days)		Laboratory			Imaging			Blood products			Rehabilitation			Rehabiliation (days)	
	Median	IQR		Median	IQR		Median	IQR		Median	IQR		Median	IQR		Mean	SD	Mean	SD	Median	IQR		Median	IQR		Median	IQR		Median	IQR		Mean	SD
Total	9500	2000	41,300	700	600	1200	0	0	4300	0	0	0	4600	900	23,500	5.1	10.3	6.3	13.0	300	100	900	200	100	400	0	0	0	0	0	0	13.5	34.8
Sex																																	
Male	11,600	2500	48,600	700	600	1500	0	0	4400	0	0	0	6100	1000	27,400	5.9	11.3	6.8	13.7	300	100	1000	200	100	400	0	0	0	0	0	0	14.6	37.3
Female	5900	1600	27,700	700	600	900	0	0	0	0	0	0	3100	300	14,400	3.5	7.6	5.4	11.5	200	0	700	200	100	400	0	0	0	0	0	0	11.1	29.0
Age																																	
16-25 years	7400	1800	42,700	700	600	3000	0	0	4300	0	0	0	3700	900	24,600	5.6	10.4	5.7	12.7	200	100	900	200	100	400	0	0	0	0	0	0	14.5	38.0
26-40 years	8900	1800	46,100	700	600	1200	0	0	4400	0	0	0	4500	900	27,300	6.0	13.2	6.3	12.7	300	100	1000	200	100	400	0	0	0	0	0	0	14.2	35.8
41-64 years	10,400	2100	44,300	700	600	1300	0	0	4300	0	0	0	5500	900	25,400	5.2	10.0	6.6	13.2	300	100	1000	200	100	400	0	0	0	0	0	0	13.8	36.8
≥65 years	10,000	2400	34,600	700	600	900	0	0	0	0	0	0	4900	900	18,600	4.1	8.4	6.4	13.2	300	100	800	200	100	400	0	0	0	0	0	0	11.9	28.8
Medical history																																	
Healthy patient	8300	1800	40,700	700	600	1200	0	0	4300	0	0	0	4300	900	23,100	5.2	9.9	5.8	12.0	300	100	900	200	100	400	0	0	0	0	0	0	13.3	34.6
Mild systemic disease	10,300	2300	39,000	700	600	1200	0	0	3800	0	0	0	5200	900	24,000	5.0	11.0	6.7	12.7	300	100	900	200	100	400	0	0	0	0	0	900	13.8	36.0
Severe systemic disease	12,100	2200	47,700	700	700	1200	0	0	4700	0	0	0	5400	900	27,200	5.1	10.3	7.7	17.9	300	100	1100	200	100	400	0	0	200	0	0	3700	13.4	32.1
Cause of injury																																	
Road traffic accident	14,800	3100	57,900	700	700	3000	0	0	4400	0	0	2000	7900	1200	32,500	6.3	10.8	6.8	12.8	400	100	1100	200	100	400	0	0	200	0	0	7100	16.8	38.4
Fall	7100	1800	30,800	700	600	900	0	0	3000	0	0	0	3700	900	16,600	4.4	10.3	5.8	12.2	200	100	800	200	100	400	0	0	0	0	0	0	11.5	32.8
Violence	5000	1500	24,200	700	400	900	0	0	5300	0	0	0	2200	900	12,800	3.4	7.3	6.1	16.7	200	100	600	300	100	400	0	0	0	0	0	0	8.9	26.4
Self-harm	43,700	14,700	108,000	800	600	3300	0	0	8700	2200	0	10,100	31,000	5700	69,900	10.9	13.2	16.9	27.9	900	200	2900	300	200	500	700	0	2300	0	0	16,000	22.5	37.0
Other	6600	1800	33,600	700	600	1200	0	0	0	0	0	0	3600	900	18800	4.3	8.8	5.8	11.9	200	100	700	200	100	400	0	0	0	0	0	0	11.1	32.0
TBI severity																																	
Mild	3800	1400	14,100	700	600	900	0	0	0	0	0	0	1700	300	7400	1.8	5.5	4.5	9.5	100	0	400	200	100	300	0	0	0	0	0	0	5.8	21.5
Moderate	37,800	14,900	74,200	800	700	3300	4200	0	8500	0	0	0	20,600	8000	47,500	8.9	10.9	10.1	13.5	800	400	1600	400	200	500	0	0	500	0	0	13,700	22.1	41.0
Severe	60,400	24,400	112,700	900	700	3700	4800	0	10,500	0	0	2200	37,200	13,900	70,400	13.5	14.7	10.3	19.2	1100	400	2000	400	200	600	0	0	900	0	0	23,700	32.6	51.7
Brain AIS																																	
Minor	1400	900	3300	700	300	900	0	0	0	0	0	0	300	300	1400	0.3	1.7	1.9	4.6	0	0	100	100	100	200	0	0	0	0	0	0	1.4	9.8
Moderate	1800	1100	4800	700	600	800	0	0	0	0	0	0	700	300	2000	0.7	3.3	2.8	11.7	100	0	200	200	100	200	0	0	0	0	0	0	2.9	14.6
Serious	4700	2100	11,600	700	600	900	0	0	0	0	0	0	2200	900	5600	1.0	3.8	5.5	9.2	200	100	400	200	100	300	0	0	0	0	0	0	6.1	21.9
Severe	26,900	13,500	53,800	800	700	3000	0	0	4800	0	0	1600	15,100	8200	33,000	7.2	9.6	8.9	13.1	600	300	1200	300	200	500	0	0	200	0	0	10,000	18.1	37.6
Critical	70,700	35,100	119,800	900	700	3700	6800	3800	12,300	0	0	1900	42,300	20,200	75,700	14.8	14.5	11.2	19.1	1200	600	2200	500	300	700	100	0	900	5200	0	25,900	34.9	52.0
Unsurvivable	7200	4200	15,700	700	700	3400	0	0	4200	0	0	0	3300	2900	5500	3.4	11.4	0.8	3.5	100	100	100	100	100	300	0	0	900	0	0	0	0.5	3.6
ISS																																	
Minor (≤16)	2400	1100	7100	700	600	900	0	0	0	0	0	0	1000	300	3700	0.8	3.5	3.3	8.5	100	0	300	200	100	300	0	0	0	0	0	0	4.1	18.8
Major (17-25)	19,000	7000	54,700	800	700	2700	0	0	6500	0	0	0	10,500	3500	31,000	6.4	12.6	7.9	12.6	500	200	1100	300	100	500	0	0	0	0	0	8300	16.9	38.0
Critically injured (>25)	51,800	20,300	99,300	900	700	3700	3800	0	8700	0	0	4200	30,100	10,500	61,000	11.5	12.6	10.5	17.6	1000	400	1900	400	200	500	0	0	900	0	0	18,700	27.2	46.7
Baseline pupillary reaction																																	
Both reacting	7800	1800	34,400	700	600	1000	0	0	0	0	0	0	4200	900	19,300	4.4	9.5	6.2	12.8	300	100	800	200	100	400	0	0	0	0	0	0	12.4	33.4
One reacting	51,600	15,000	102,700	900	700	3700	5300	0	10,400	0	0	1800	28,800	6200	58,600	11.0	12.1	9.1	14.7	900	300	1900	400	200	500	0	0	900	0	0	22,600	30.0	49.8
Non-reacting	31,500	8100	87,800	900	700	3700	4800	0	9600	0	0	1600	18,300	3300	54,900	10.6	15.2	6.3	14.5	400	100	1500	300	100	500	200	0	1400	0	0	7500	18.0	39.9
CT abnormalities																																	
Any CT abnormality																																	
Absent	2400	1100	10,000	700	600	900	0	0	0	0	0	0	900	300	4500	2.3	9.1	3.7	11.0	100	0	300	100	100	200	0	0	0	0	0	0	6.7	26.1
Present	23,300	7200	66,600	800	700	3100	0	0	5600	0	0	0	12,800	3700	39,600	7.4	10.7	8.5	14.1	600	200	1300	300	200	500	0	0	300	0	0	10,300	19.1	39.7
Cisternal compression	47,500	15,900	106,100	900	700	3300	6800	3300	12,300	0	0	0	27,000	7200	63,300	11.4	12.2	9.3	16.2	800	300	1900	400	200	600	0	0	1200	0	0	18,700	28.2	51.6
Midline shift	42,100	14,900	93,700	900	700	3300	6800	3800	12,300	0	0	0	23,400	6500	54,900	10.0	11.4	9.1	15.3	800	300	1700	400	200	500	0	0	1000	0	0	17,600	27.0	49.3
Subarachnoid heamorrhage	30,400	9900	74,000	800	700	3300	0	0	7700	0	0	0	16,500	5100	46,300	8.4	11.0	9.3	15.5	700	300	1500	400	200	500	0	0	400	0	0	12,900	21.6	42.5
Epidural hematoma	27,800	10900	74,400	900	700	3000	4400	0	9300	0	0	0	16,000	5900	45,600	8.6	11.7	9.5	16.8	700	300	1500	400	300	500	0	0	300	0	0	9900	19.8	43.1
Acute subdural hematoma	31,400	9400	75,900	800	700	3100	3400	0	9200	0	0	0	17,000	4900	46,400	8.6	11.0	9.1	15.5	700	300	1500	400	200	500	0	0	500	0	0	13,200	21.8	42.8
Diffuse axonal injury	42,300	10,800	96,400	900	700	3700	0	0	7000	0	0	2200	24,200	5400	57,500	10.2	12.2	9.1	17.0	800	300	1700	400	200	600	0	0	400	0	0	17,500	26.7	46.8
Contusion	33,300	11,400	86,500	800	700	3300	3500	0	8700	0	0	0	20,100	6400	51,800	9.5	11.7	9.4	15.7	800	300	1600	400	300	600	0	0	400	0	0	13,800	23.0	43.7
In-hospital mortality																																	
No	8500	1800	42,000	700	600	1200	0	0	3500	0	0	0	4500	900	23,500	5.1	10.4	6.8	13.4	300	100	900	200	100	400	0	0	0	0	0	1800	14.6	36.0
Yes	18,900	7200	38,300	700	700	3400	4200	0	8700	0	0	0	8000	3300	24,300	5.9	9.2	0.6	3.1	200	100	600	300	100	400	0	0	1200	0	0	0	-	-
GOSE-6 months disability																																	
1	23,700	7900	52,200	700	700	3300	3800	0	8700	0	0	0	11,900	3300	34,900	7.5	14.2	4.3	11.7	300	100	1000	300	100	500	0	0	800	0	0	0	5.1	18.1
2-3	94,300	41,200	155,800	900	700	3500	5200	0	12,300	0	0	2500	52,700	21,600	88,600	16.3	14.3	17.8	27.5	1700	800	3000	400	200	700	0	0	1100	16,300	0	46,000	61.3	70.8
4	45,100	12,400	110,700	900	700	3400	0	0	7000	0	0	3000	28,800	7200	63,500	10.7	12.1	12.3	18.2	1000	400	2000	400	200	500	0	0	600	0	0	22,900	28.8	44.9
5	36,000	10,400	75,500	800	700	3400	0	0	6800	0	0	1800	18,300	5200	42,600	8.2	12.3	9.4	11.1	800	300	1500	300	200	500	0	0	300	0	0	17,500	23.2	35.6
6	17,100	4900	49,900	700	700	3000	0	0	4100	0	0	1900	8700	2200	28,100	5.4	9.0	7.2	9.6	400	200	1100	300	100	500	0	0	0	0	0	9400	15.9	30.8
7	5400	1800	18,900	700	600	900	0	0	0	0	0	0	2900	900	11,300	2.4	5.9	4.7	6.7	200	100	600	200	100	400	0	0	0	0	0	0	6.3	18.5
8	2600	1200	8700	700	600	900	0	0	0	0	0	0	1200	300	4800	1.4	4.8	3.3	8.1	100	0	300	200	100	300	0	0	0	0	0	0	3.3	15.8

ICU, intensive care unit; IQR, interquartile range; SD, standard deviation; TBI, traumatic brain injury; AIS, Abbreviated Injury Score; ISS, Injury Severity Score; CT, computed tomography; GOSE, Glasgow Outcome Scale Extended

### Healthcare costs

Median intramural healthcare costs for mild, moderate, and severe TBI patients in Europe were, respectively, €3,800 [IQR €1,400–€14,000], €37,800 [IQR €14,900–€74,200], and €60,400 [IQR €24,400–€112,700], with males (€11,600; IQR [€2,500–€48,600]) having higher costs than females (€5,900; IQR [€1,600–€27,600]) (Table 3). A similar increase in costs was found for increasing systemic injury severity: minor injury (ISS ≥ 16) €2,400 [IQR €1,100–€7,100], major injury (ISS 17–25), €19,000 [IQR €7,000–€54,700], and critically injured (ISS >25) €51,800 [IQR €20,300–€99,200]. The costs for patients 16–25 years of age, 26–40 years of age, 41–64 years of age, and ≥65 years of age were, respectively, €7,400 [IQR €1,800–€42,700], €8,900 [IQR €1,800–€46,100], €10,400 [IQR €2,200–€44,300], and €10,000 [IQR €2,400–€34,600]. Across all severities, costs increased with age. Although elderly patients (≥ 65 years) had shorter ICU LOS and lower costs for surgical interventions, they had longer ward LOS (Supplementary Table S5). A worse pre-morbid overall health state was accompanied by higher costs in mild and moderate TBI patients, whereas costs were lower for severe TBI. Patients with CT abnormalities had higher costs than patients without CT abnormalities. Self-harm €43,700 [IQR €15,000–€107,000] and road traffic incidents €14,800 [IQR €3,100–€57,900], as causes of injury also showed high costs. Patients with mild TBI who died during hospital admission had higher median costs than survivors (€3,800 vs. €14,300). In contrast, patients surviving hospital admission after moderate (€42,000 vs. €22,800) and severe TBI (€75,800 vs. €19,400) had higher costs than patients who died during admission. Mean costs are available in Supplementary Tables S5 and S6.

**Table 3.** Median Intramural Costs of Traumatic Brain Injury (TBI) According to Trauma Severity

Patient characteristics	TBI severity																
	Mild				Moderate				Severe				Total				
	Median (€)	IQR			Median (€)	IQR			Median (€)	IQR			Median (€)	IQR			*p* value
Total	3800	1400	-	14,100	37,800	14,900	-	74,200	60,400	24,400	-	112,400	9500	2000	-	41,300	<0.001
Sex																	
Male	4400	1800	-	15,400	40,800	15,100	-	78,800	64,100	27,800	-	115,000	11,600	2500	-	48,600	<0.001
Female	2900	1200	-	11,300	33,200	14,900	-	70,100	52,100	19,200	-	103,100	5900	1600	-	27,600	<0.001
Age																	
16-25 years	2900	1400	-	8500	31,900	8700	-	81,000	71,500	26,600	-	121,300	7400	1800	-	42,700	<0.001
26-40 years	2500	1100	-	10,500	41,200	14,900	-	86,300	74,700	35,100	-	121,000	8900	1800	-	46,100	<0.001
41-64 years	4000	1500	-	14,000	44,500	20,800	-	75,100	64,700	31,300	-	114,300	10,400	2200	-	44,300	<0.001
≥65 years	5400	1800	-	20,100	32,900	14,700	-	60,700	34,500	10,500	-	72,900	10,000	2400	-	34,600	<0.001
Medical history																	
Healthy patient	3400	1400	-	11,300	37,800	14,500	-	78,100	65,700	28,100	-	114,100	8300	1800	-	40,700	<0.001
Mild systemic disease	4400	1500	-	16,800	34,700	18,800	-	66,600	57,200	19,800	-	112,900	10,300	2300	-	39,000	<0.001
Severe systemic disease	4900	1400	-	21,900	44,200	10,900	-	84,000	52,500	18,300	-	91,600	12,100	2200	-	47,500	<0.001
Cause of injury																	
Road traffic accident	4700	1800	-	15,900	44,000	21,100	-	73,100	69,900	31,600	-	113,800	14,800	3100	-	57,900	<0.001
Fall	3400	1400	-	13,800	30,600	12,400	-	74,300	50,100	18,400	-	103,600	7100	1800	-	30,800	<0.001
Violence	2500	1100	-	9500	30,400	14,900	-	61,400	77,300	42,100	-	138,700	5000	1500	-	24,200	<0.001
Self-harm	19,400	7000	-	43,700	97,100	47,800	-	169,000	52,100	17,000	-	110,200	43,700	15,000	-	107,000	0.037
Other	3100	1100	-	10,500	24,600	9000	-	55,800	59,800	26,800	-	110,300	6600	1800	-	32,900	<0.001
Brain AIS																	
Minor	1400	900	-	3200	10,000	3300	-	16,600	12,600	3200	-	30,900	1400	900	-	3300	<0.001
Moderate	1800	1100	-	4400	4500	2000	-	8300	28,900	5500	-	79,200	1800	1100	-	4800	<0.001
Serious	4300	2100	-	10,600	9600	4100	-	30,100	15,900	6400	-	58,600	4700	2100	-	11,600	<0.001
Severe	18,700	10,800	-	36,100	34,700	17,400	-	60,500	48,600	24,000	-	89,200	26,900	13,600	-	53,600	<0.001
Critical	58,800	28,600	-	96,700	57,500	29,100	-	107,300	76,200	39,600	-	125,900	70,700	35,100	-	119,700	<0.001
Unsurvivable	20,400	7700	-	30,500	4200	2900	-	5700	7200	4200	-	15,000	7200	4200	-	15,500	0.080
ISS																	
Minor (≤16)	2000	1100	-	5600	13,900	7400	-	30,800	24,600	6900	-	49800	2400	1100	-	7100	<0.001
Major (17-25)	10,100	4000	-	22,700	41,900	25,200	-	77,400	58,600	24,300	-	114,200	19,000	7000	-	54,700	<0.001
Critically injured (>25)	29,100	13400	-	63,400	54,000	27,200	-	90,600	66,900	28,400	-	114,400	51,800	20,300	-	99,200	<0.001
Baseline pupillary reaction																	
Both reacting	3800	1400	-	13,800	35,200	14,900	-	72,000	64,600	31,200	-	113,900	7800	1800	-	34,400	<0.001
One reacting	8300	2600	-	29,300	59,800	36,600	-	79,700	69,900	35,400	-	131,600	51,600	15,100	-	102,600	<0.001
Non-reacting	5700	1000	-	35,700	45,100	9600	-	84,100	36,500	10,900	-	89,100	31,500	8300	-	87,400	<0.001
CT abnormalities																	
Any CT abnormality																	
Absent	1800	1100	-	5100	27,200	7700	-	57,000	47,000	17,000	-	106,900	2400	1100	-	10,000	<0.001
Present	10,400	3900	-	26,100	42,000	17,100	-	78,000	63,200	27,600	-	113,400	23,300	7200	-	66,600	<0.001
Cisternal compression	31,100	13300	-	72,500	43,100	15,400	-	86,800	57,400	20,100	-	119,100	47,500	16,000	-	106,100	<0.001
Midline shift	30,200	11900	-	68,200	39,400	14,700	-	72,100	52,100	19,900	-	110,500	42,100	15,000	-	93,600	0.003
Subarachnoid hemorrhage	13,500	5000	-	32,800	42,000	19,100	-	84,400	64,500	28,400	-	113.900	30,400	9900	-	74,000	<0.001
Epidural hematoma	14,200	6500	-	31,700	37,100	15,800	-	64,100	73,300	33,300	-	125,200	27,800	10,900	-	74,400	<0.001
Acute subdural hematoma	12,400	5100	-	34,400	45,100	19,200	-	88,500	60,200	24,100	-	115.500	31,400	9400	-	75,900	<0.001
Diffuse axonal injury	9700	3600	-	21,800	48,800	27,200	-	93,800	82,600	48,000	-	119.000	42,300	11,000	-	96,300	<0.001
Contusion	14,400	5900	-	34,200	45,200	21,000	-	86,800	70,700	32,400	-	123,600	33,300	11,400	-	86,500	<0.001
Inhospital mortality																	
No	3800	1400	-	13,800	42,000	16,700	-	78,600	75,800	38,700	-	126,700	8500	1800	-	42,000	<0.001
Yes	14,300	1600	-	34,500	22,800	7800	-	32,400	19,400	8200	-	40,800	18,900	7200	-	37,900	0.069
GOSE-6 months disability																	
1	13,600	2400	-	33,400	29,500	11,500	-	54,700	25,500	10,200	-	58,000	23,700	8000	-	52,200	<0.001
2-3	38,000	11100	-	96,800	79,100	46,900	-	151,200	128,600	77,700	-	177,800	94,300	41,600	-	155,300	<0.001
4	13,900	4000	-	38,400	56,600	32,200	-	114,300	110,500	69,000	-	142,300	45,100	12,400	-	110,600	<0.001
5	12,900	4000	-	35,300	46,500	25,900	-	78,600	81,300	51,300	-	116,500	36,000	10,500	-	75,200	<0.001
6	8600	3000	-	27,000	41,800	17,100	-	78,600	64,500	36,000	-	98,600	17,100	4900	-	49,700	<0.001
7	3400	1800	-	11,100	24,000	13,500	-	58,800	38,700	21,600	-	78,800	5400	1800	-	18,900	<0.001
8	2200	1100	-	6000	26,300	9400	-	46,000	32,700	11,600	-	71,200	2600	1200	-	8700	<0.001

The *p* value assesses the null hypothesis of no differences among the mild, moderate, and severe subgroups.

IQR, interquartile range; AIS, Abbreviated Injury Score; ISS, Injury Severity Score; CT, computed tomography; GOSE, Glasgow Outcome Scale. Extended

### Sex differences in intramural costs

**FIG. 2.**
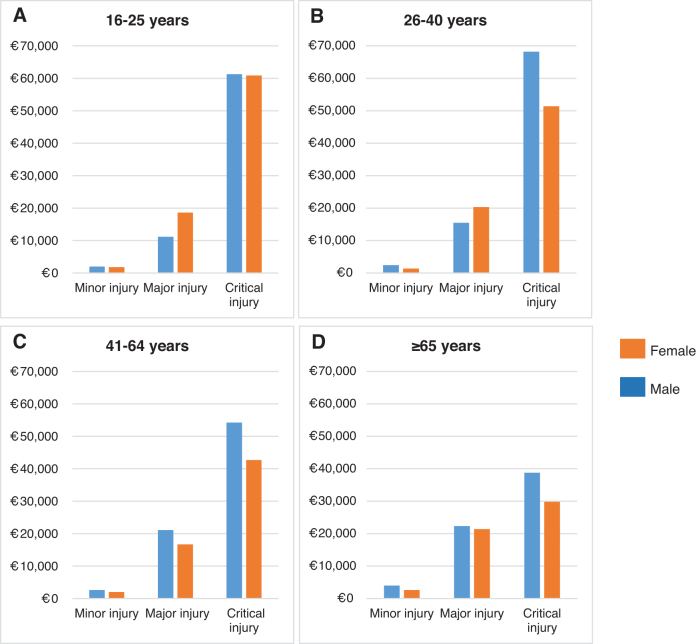
The median total intramural costs for male and female patients are plotted according to injury severity and age category. The injury severity was determined using the baseline systemic Injury Severity Score (ISS) and was categorized into three groups: ISS ≤16 (minor injury); ISS 17–25 (major injury); ISS >25 (critical injury). The four panels represent the four different age categories: **(A)** 16–25 years, **(B)** 26–40 years, **(C)** 41–64 years, and **(D)** ≥ 65 years.

Male patients (median €11,600 [IQR €2,500–€48,600]) had higher median costs than female TBI patients (median €5,900 [IQR €1,600–€27,600]) (Table 3). Male patients incurred higher costs, across almost all age groups and injury severities (Fig. 2). Male patients showed higher costs across all seven intramural cost categories (*p* < 0.001). ICU LOS (mean 5.9 vs. 3.5 days) and ward LOS (mean LOS 6.8 vs. 5.4 days) were both longer for male than for female patients (*p* < 0.001) (Table 2). Irrespective of adjustment for several patient characteristics, costs remained higher for male patients (Table 4).

**Table 4.** Associations With Total Healthcare Costs Based on Generalized Linear Models

Patient characteristics	Generalized linear model									
	Multi-variate univariable					Multi-variate multivariable				
	Exp[β]	95% CI			*p *value	Exp[β]	95% CI			*p* value
Sex										
Male	(ref)					(ref)				
Female	0.72	0.66	-	0.78	<0.001	0.80	0.75	-	0.85	<0.001
Age										
16-25 years	(ref)					(ref)				
26-40 years	1.04	0.91	-	1.19	0.547	1.13	1.02	-	1.24	0.015
41-64 years	1.03	0.92	-	1.16	0.580	1.04	0.96	-	1.14	0.353
≥65 years	0.89	0.79	-	1.01	0.074	1.13	1.01	-	1.25	0.026
Medical history										
Healthy patient	(ref)					(ref)				
Mild systemic disease	0.97	0.89	-	1.07	0.572	1.06	0.99	-	1.14	0.105
Severe systemic disease	1.09	0.96	-	1.25	0.198	1.28	1.15	-	1.42	<0.001
Cause of injury										
Road traffic accident	(ref)					(ref)				
Fall	0.76	0.70	-	0.83	<0.001	0.84	0.78	-	0.89	<0.001
Violence	0.73	0.63	-	0.85	<0.001	0.94	0.85	-	1.05	0.291
Self-harm	0.67	0.56	-	0.80	<0.001	0.75	0.66	-	0.85	<0.001
Other	1.83	1.26	-	2.68	0.002	1.09	0.83	-	1.43	0.536
TBI severity										
Mild	(ref)					(ref)				
Moderate	3.52	3.10	-	3.99	<0.001	1.46	1.31	-	1.63	<0.001
Severe	4.88	4.48	-	5.32	<0.001	1.67	1.52	-	1.84	<0.001
Brain AIS										
Minor	(ref)					(ref)				
Moderate	1.80	1.60	-	2.03	<0.001	1.30	1.17	-	1.44	<0.001
Serious	2.84	2.58	-	3.13	<0.001	1.61	1.46	-	1.77	<0.001
Severe	9.79	8.77	-	10.93	<0.001	2.75	2.43	-	3.13	<0.001
Critical	17.70	15.99	-	19.59	<0.001	2.75	2.37	-	3.19	<0.001
Unsurvivable	3.79	3.00	-	4.79	<0.001	0.39	0.31	-	0.51	<0.001
ISS										
Minor (≤16)	(ref)					(ref)				
Major (17-25)	4.51	4.12	-	4.94	<0.001	1.85	1.70	-	2.01	<0.001
Critically injured (>25)	7.10	6.55	-	7.70	<0.001	2.57	2.34	-	2.81	<0.001
Hypoxia										
No	(ref)					(ref)				
Yes	2.08	1.74	-	2.50	<0.001	1.15	1.00	-	1.32	0.045
Hypotension										
No	(ref)					(ref)				
Yes	2.32	1.96	-	2.76	<0.001	1.18	1.03	-	1.35	0.016
Hemoglobin	0.81	0.80	-	0.82	<0.001	0.91	0.90	-	0.93	<0.001
Glucose	1.15	1.12	-	1.17	<0.001	1.04	1.03	-	1.06	<0.001
Marshall CT classification										
1	(ref)					(ref)				
2	4.05	3.74	-	4.40	<0.001	1.53	1.39	-	1.69	<0.001
3	8.03	6.68	-	9.65	<0.001	2.17	1.78	-	2.66	<0.001
4	5.96	4.05	-	8.79	<0.001	2.40	1.72	-	3.35	<0.001
5	9.93	6.59	-	14.97	<0.001	2.49	1.77	-	3.49	<0.001
6	7.11	6.43	-	7.87	<0.001	2.34	2.05	-	2.67	<0.001
CT abnormalities										
Cisternal compression	2.55	2.29	-	2.85	<0.001	0.94	0.81	-	1.08	0.394
Midline shift	2.19	1.92	-	2.48	<0.001	0.86	0.74	-	1.00	0.044
Subarachnoid heamorrhage	2.65	2.45	-	2.87	<0.001	1.03	0.95	-	1.13	0.444
Epidural hematoma	1.59	1.39	-	1.82	<0.001	0.98	0.89	-	1.08	0.654
Acute subdural hematoma	2.11	1.93	-	2.31	<0.001	1.18	1.09	-	1.28	<0.001
Diffuse axonal injury	1.92	1.69	-	2.19	<0.001	0.98	0.90	-	1.06	0.623
Contusion	2.46	2.27	-	2.68	<0.001	0.94	0.85	-	1.04	0.259

CI, confidence interval; TBI, traumatic brain injury; AIS, Abbreviated Injury Score; ISS, Injury Severity Score; CT, computed tomography.

### Between-country differences in healthcare consumption

**FIG. 3.**
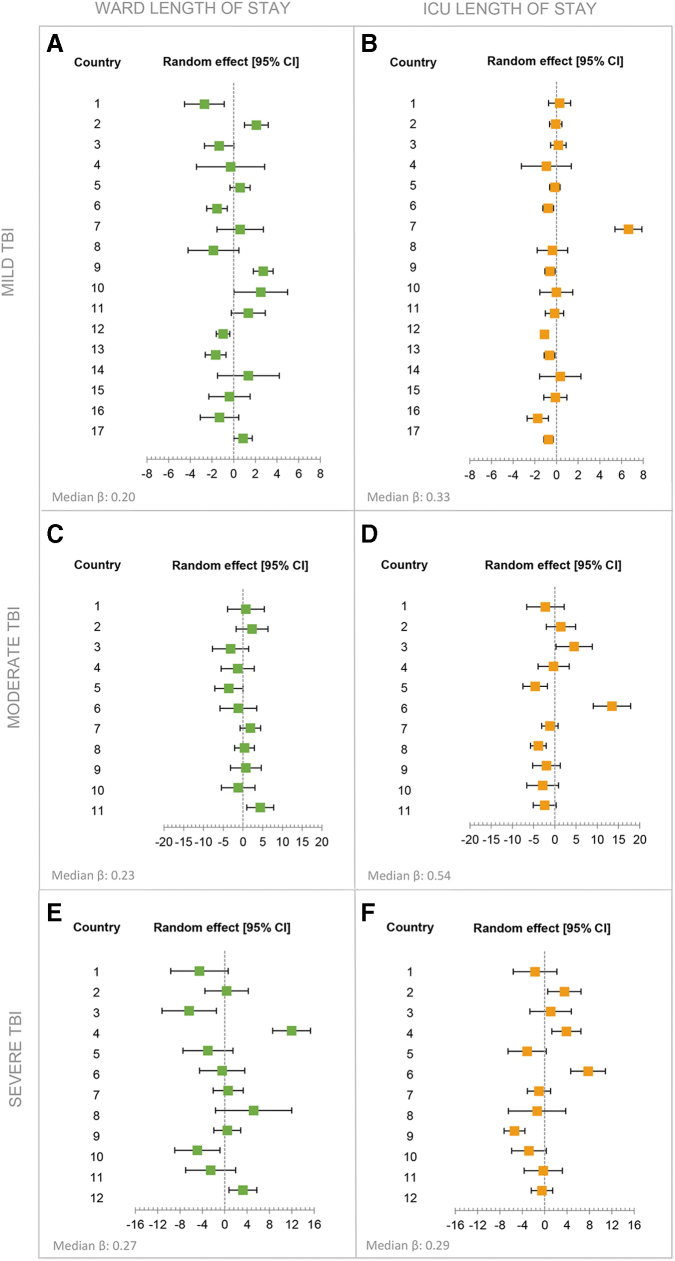
This panel shows forest plots reporting the random country effect (random intercept estimate and 95% confidence intervals) on the length of stay at the ICU and ward for mild **(A–B)**, moderate **(C–D)** and severe **(E–F)** TBI patients. Countries including fewer than five patients per severity group were excluded from this analysis. The models included adjustment according to the International Mission for Prognosis and Analysis of Clinical Trials in TBI (IMPACT) prognostic model. The median β reflects the between-country variation; a median β equal to 0 represents no variation, the larger the median β, the larger the variation.

Case-mix of patients varied substantially among countries. The total number of patients per country ranged from 15 to 962. France (52%), Sweden (35%), and Lithuania (33%) had a high percentage of severe TBI patients. Patients with critical injury (Injury Severity Score [ISS] = critical) were mostly found in France (67%), Italy (42%) and the United Kingdom (37%) (Supplementary Table S7). Throughout Europe, costs related to hospitalization were the largest contributor to the total intramural costs, especially in Romania (83%), Austria (76%), and France (72%) (Supplementary Fig. S1). The costs generated from intracranial surgery were the highest in Denmark (12%), Lithuania (12%), Sweden (13%), and Hungary (13%). The multi-variable linear regression model showed that across all TBI severities and adjusted for patient characteristics, some differences among countries in the LOS in the ICU and on the ward were present (Fig. 3A–3F). Most profound differences were visible in the LOS in the ICU, especially in the moderate and severe patient groups (Fig. 3D and 3F). Outliers within this analysis are most profoundly caused by the selective sampling of countries. The median β value indicates that mild, moderate, and severe TBI patients with the same baseline characteristics from a random country will have an average ICU LOS longer by 0.33 days, 0.54 days, and 0.29 days, respectively, when compared with another random country (Fig. 3A–F).

### Generalized linear model

Female patients showed lower total intramural costs with an OR of 0.80 [CI 0.75–0.85] times lower than male patients. Increasing TBI severity was associated with higher costs for moderate and severe patients: OR 1.46 [CI 1.31–1.63] and OR 1.67 [CI 1.52–1.84], respectively. Compared with minor brain AIS, severe and critically injured patients showed higher costs (OR 2.75 [CI 2.43–3.13] and 2.75 [CI 2.37–3.19]) (Table 4). Hypotension at admission was also associated with higher costs with an OR of 1.18 [CI 1.03–1.35]. Increasing severity of CT abnormalities, as measured by the Marshall CT score, was also associated with higher costs.

## Discussion

The median intramural healthcare costs of a TBI patient in Europe were €3,800 [IQR €1,400–€14,000] for mild, €37,800 [IQR €14,900–€74,200] for moderate, and €60,400 [IQR €24,400–€112,700] for severe TBI. Costs generally increased with higher age, higher injury severity, and male gender. For all TBI severity groups and across all countries, hospitalization was the main driver for total intramural costs.

### Patient population

Studies describing the global burden of TBI, estimated that mild TBI accounted for 81% of injuries, moderate TBI for 11% and severe TBI for 8% and estimated that the first-year lifetime costs per person for mild TBI was between US$3395 and US$4636 and respectively US$21379 and US$36648 for moderate and severe patients.^20,39^ In comparison to these studies, the CENTER-TBI population included only those patients with a CT indication and recruited mostly patients from academic medical centers, leading to a lower proportion of mild TBI patients and higher rates of severely injured patients. Severe TBI patients have longer LOS and undergo more neurosurgical interventions compared to the other severity levels of TBI, which could result in higher total intramural costs for the entire CENTER-TBI population.^17,20,40–44^ The exclusion of TBI patients without a CT indication combined with higher proportions of severely injured patients show that the CENTER-TBI study is not fully representative of the European TBI population.

As mentioned, the European TBI population is composed mostly of mild TBI patients, for whom CT is not always indicated, and neurosurgical interventions are required in <1%.^45^ Notwithstanding, stratification on injury severity in our study was based on the baseline clinical assessment wherein clinical deterioration was not accounted for. Additionally, the mild TBI population is a highly heterogeneous group, and although classified as mild, ∼50% do not reach full recovery 6 months after injury. The possibility of clinical deterioration combined with the heterogeneity of this population and possible presence of extracranial injury could explain their comparable need for inpatient rehabilitation and the observed inhospital mortality rate.^46^

### Sex differences

We showed that male patients incurred higher total intramural costs, in almost all age and severity groups, than female patients. It is known that TBI most commonly affects younger adults, specifically men, causing substantial costs to society as a result of their death and disability.^47–49^ Common causes of trauma within the younger male population are road traffic incidents and interpersonal violence, mostly resulting in severe TBI and concomitant severe injury to the chest, abdomen, and extremities.^50–52^ Compared with patients with isolated TBI, defined as brain injury without concomitant severe extracranial injury, patients with severe extracranial injury have longer hospitalizations because of the necessity of continuing treatment for body sites other than the head.^53^ The presence of severe extracranial injury could lead to longer hospital LOS resulting in higher intramural costs and causing differences in costs between males and females. However, higher costs for male patients remained after adjustment for relevant confounders, including extracranial injury. Several studies have shown that in comparison to male TBI patients, female TBI patients have lower access to trauma centers and are less often admitted to the ICU. Regarding TBI guideline adherence, CT seems to be performed less often in women than in men.^54–56^ Within CENTER-TBI, differences in care pathways were most frequently observed in patients who sustained mild TBI, wherein women with comparable injury severity and demographic characteristics were more likely to be discharged home after presenting to the ER and were less likely to be admitted to the ICU.^56^ The differences in healthcare consumption and costs between males and females could therefore be explained by differences in management of TBI and suboptimal healthcare access among female TBI patients.

### The elderly and TBI

We reported that an increase in age is associated with an increase in costs, which is line with previous studies showing that increasing age, severe brain injury, and extracranial injury are related to higher hospital costs.^41,57^ The cost pattern of the elderly did however, differ from the younger patient group, as they had shorter ICU LOS and lower costs for surgical interventions. The difference in healthcare consumption by the elderly could be explained by (1) mechanism of injury and (2) their pre-morbid health state.

In the elderly population, low energy falls are a common cause of TBI, which are most commonly adjoined by injuries to the lower extremities. Although these injuries are expected to incur higher costs, the need for critical care or emergency interventions remains low.^49,58–60^ Additionally, although most older patients initially had mild TBI, proportions of in-hospital mortality remained high.^61^ Because of vulnerability and pre-existing comorbidities, older adults are less likely to survive their TBI than are their younger counterparts, which could presumably lead to higher consumption of care during the end phase life.^61,62^

### Between-country differences in healthcare consumption

In this study, we found some differences in LOS of TBI patients in the ICU and on the ward across countries. Although part of this difference could be explained by a different case mix of patients in each country, differences in ward and ICU LOS remained within each TBI severity level. When interpreting these differences, we should acknowledge that the design of CENTER-TBI, with enrollment of patients in three admission strata (ER, ward, and ICU) led to different recruitment procedures of TBI severities among countries (i.e. some countries enrolled only patients in the ICU stratum, meaning patients admitted directly to the ICU upon presentation). Although we performed extensive case-mix adjustment, we cannot exclude the possibility of remaining differences in the patient population. Besides differences in patient population, the observed between-country differences in healthcare consumption can still be for other reasons, such as the overall health status of the residential population, the proportion of patients with insurance, pharmaceutical costs, and personnel costs.^63^ Additionally, the economic development of a country determines the health spending per person.^64^ In general, differences in expenditure also affect the outcome of TBI patients, as lower- resource, developing countries experience significant higher mortality rates than the higher-resource countries.^65^ Using GDP-corrected prices, we have adjusted for this factor within this study. In addition to these economic factors, the organization of care and guidelines adaptation is an important key factor in healthcare expenditure. The difference in organization of care can result in a difference of guidelines being used; for example, it is known that some countries are more likely to perform CT scans in patients with mild TBI.^54,66^ Within TBI care, clinical guidelines are scarce and adherence is suboptimal, resulting in considerable between-country variation in treatment of TBI and subsequently different expenditure patterns across countries.^54,67^ A previous study has shown that there is considerable variation regarding ICU admission policies, especially in the mild TBI population, wherein it is unclear whether a liberal admission policy is truly benefiting the patient while costs are rising.^68^

### Strength and limitations

The most important strength of this study is the availability of detailed data of high quality collected from several European countries. The data provide a detailed perspective for all severities of TBI, including data about different age groups with detailed clinical presentation, neuroimaging, and performed interventions. However, several limitations should be acknowledged. The CENTER-TBI study consisted mostly of trauma levels I and II hospitals, resulting in a population of relatively severely injured patients. This may not correctly represent the total TBI population in Europe, as trauma level I centers are known to have overall higher expenses resulting in higher costs.^69^ This, combined with the selective sampling per country, makes it overall difficult to interpret between-country differences.

Total costs were calculated using inflation- and GDP-corrected cost prices, as health financial systems are determinative of the care products` cost prices. Because of the use of inflation- and GDP-corrected prices in this study, we were able to compare the cost of TBI across countries, and focus on healthcare consumption rather than price differences. However, it should be noted that adjustment for GDP-PPP does not fully compensate for actual cost differences among countries. Second, our study did not include detailed information about the interventions in the first hospital for referred patients, despite the burden of TBI in acute care being substantial.^11^ With 17% of our study population consisting of secondary referrals, missing data on the total healthcare consumption in acute care setting at the referring hospital, could cause an underestimation of the total costs.

In our study, information on long-term healthcare consumption, such as outpatient rehabilitation care and outpatient clinic visits, was not available. Outpatient rehabilitation care and outpatient clinic visits are inevitably large contributors to the overall costs of TBI. After TBI, a range of problems can persist, including cognitive impairment, post-concussion symptoms, emotional difficulties, and functional limitations, requiring long-term outpatient care.^46^ A study conducted in the United States has shown that patients receiving inpatient rehabilitation still experience major health consequences 5 years after injury, wherein 12% were living in an institutional setting and almost 50% were readmitted to the hospital at least once.^70^ A study from New Zealand showed that in the first year after trauma, patients use their general practitioner in 36% of the cases, allied health in 18% of cases, and specialized services in 14% of cases, increasing respectively with TBI severity.^20^ In our study, we observed that inpatient rehabilitation accounted for 19% of the total costs across all TBI severities. This is most probably an underestimated contribution to the total costs, as a previous study has shown that the need for rehabilitation services is largely unmet within the TBI population.^71^ We should additionally acknowledge that the long-term consequences of TBI are the drivers of the indirect costs caused by loss of productivity, disability, and reduced quality of life.^46^ These indirect costs are contemplated to be the largest contributors to the overall costs related to TBI, indicating that the economic impact of TBI is even higher than shown in this study.

### Recommendations

Intramural costs of TBI are significant, with hospital admission being the largest contributor. Costs increased with trauma severity, male patients incurred higher costs, and cost patterns of the elderly differed from those of the overall TBI population. This knowledge about healthcare expenses could be a leading step toward more cost-efficient TBI care. Hospitalization (ICU LOS in particular), incurs the highest costs and differs among countries. Improvements in resource allocation and eventual reduction of costs could be effected by the development of admission guidelines wherein only those who would truly benefit will be admitted to the ICU, combined with special attention to gender differences in assessment of patients. A leading step toward tailored and cost-effective TBI treatment, is, for example, the use of acute serum biomarkers to determine CT indication in mild TBI patients, thereby preventing unnecessary imaging.^72^ Additionally, discharge planning according to patient needs and preventive interventions targeting in-hospital complications are highly valuable in reducing unnecessary healthcare consumption. The long-term consequences of TBI are of substantial concern for the patient, the healthcare provider, and, eventually, society. Advanced care planning, wherein patients start early on with rehabilitation, could lead to reduction of hospitalization and better patient outcome, which will subsequently lead to a reduction of the indirect costs related to TBI. Differences in healthcare consumption between males and females should also be explored more extensively, as differences in the management of TBI could also lead to different outcomes. Conclusively, TBI patients must be considered as a distinct patient population, with targeted interventions that suit the different subgroups within TBI, in order to reduce costs.

## Supplementary Material

Supplemental data

## CENTER-TBI Participants and Investigators

Cecilia Åkerlund,^1^ Krisztina Amrein,^2^ Nada Andelic,^3^ Lasse Andreassen,^4^ Audny Anke,^5^ Anna Antoni,^6^ Gérard Audibert,^7^ Philippe Azouvi,^8^ Maria Luisa Azzolini,^9^ Ronald Bartels,^10^ Pál Barzó,^11^ Romuald Beauvais,^12^ Ronny Beer,^13^ Bo-Michael Bellander,^14^ Antonio Belli,^15^ Habib Benali,^16^ Maurizio Berardino,^17^ Luigi Beretta,^9^ Morten Blaabjerg,^18^ Peter Bragge,^19^ Alexandra Brazinova,^20^ Vibeke Brinck,^21^ Joanne Brooker,^22^ Camilla Brorsson,^23^ Andras Buki,^24^ Monika Bullinger,^25^ Manuel Cabeleira,^26^ Alessio Caccioppola,^27^ Emiliana Calappi,^27^ Maria Rosa Calvi,^9^ Peter Cameron,^28^ Guillermo Carbayo Lozano,^29^ Marco Carbonara,^27^ Simona Cavallo,^17^ Giorgio Chevallard,^30^ Arturo Chieregato,^30^ Giuseppe Citerio,^31,32^ Hans Clusmann,^33^ Mark Coburn,^34^ Jonathan Coles,^35^ Jamie D. Cooper,^36^ Marta Correia,^37^ Amra Čović,^38^ Nicola Curry,^39^ Endre Czeiter,^24^ Marek Czosnyka,^26^ Claire Dahyot-Fizelier,^40^ Paul Dark,^41^ Helen Dawes,^42^ Véronique De Keyser,^43^ Vincent Degos,^16^ Francesco Della Corte,^44^ Hugo den Boogert,^10^ Bart Depreitere,^45^ Đula Đilvesi,^46^ Abhishek Dixit,^47^ Emma Donoghue,^22^ Jens Dreier,^48^ Guy-Loup Dulière,^49^ Ari Ercole,^47^ Patrick Esser,^42^ Erzsébet Ezer,^50^ Martin Fabricius,^51^ Valery L. Feigin,^52^ Kelly Foks,^53^ Shirin Frisvold,^54^ Alex Furmanov,^55^ Pablo Gagliardo,^56^ Damien Galanaud,^16^ Dashiell Gantner,^28^ Guoyi Gao,^57^ Pradeep George,^58^ Alexandre Ghuysen,^59^ Lelde Giga,^60^ Ben Glocker,^61^ Jagoš Golubovic,^46^ Pedro A. Gomez,^62^ Johannes Gratz,^63^ Benjamin Gravesteijn,^64^ Francesca Grossi,^44^ Russell L. Gruen,^65^ Deepak Gupta,^66^ Juanita A. Haagsma,^64^ Iain Haitsma,^67^ Raimund Helbok,^13^ Eirik Helseth,^68^ Lindsay Horton,^69^ Jilske Huijben,^64^ Peter J. Hutchinson,^70^ Bram Jacobs,^71^ Stefan Jankowski,^72^ Mike Jarrett,^21^ Ji-yao Jiang,^58^ Faye Johnson,^73^ Kelly Jones,^52^ Mladen Karan,^46^ Angelos G. Kolias,^70^ Erwin Kompanje,^74^ Daniel Kondziella,^51^ Evgenios Kornaropoulos,^47^ Lars-Owe Koskinen,^75^ Noémi Kovács,^76^ Ana Kowark,^77^ Alfonso Lagares,^62^ Linda Lanyon,^58^ Steven Laureys,^78^ Fiona Lecky,^79,80^ Didier Ledoux,^78^ Rolf Lefering,^81^ Valerie Legrand,^82^ Aurelie Lejeune,^83^ Leon Levi,^84^ Roger Lightfoot,^85^ Hester Lingsma,^64^ Andrew I.R. Maas,^43^ Ana M. Castaño-León,^62^ Marc Maegele,^86^ Marek Majdan,^20^ Alex Manara,^87^ Geoffrey Manley,^88^ Costanza Martino,^89^ Hugues Maréchal,^49^ Julia Mattern,^90^ Catherine McMahon,^91^ Béla Melegh,^92^ David Menon,^47^ Tomas Menovsky,^43^ Ana Mikolic,^64^ Benoit Misset,^78^ Visakh Muraleedharan,^58^ Lynnette Murray,^28^ Ancuta Negru,^93^ David Nelson,^1^ Virginia Newcombe,^47^ Daan Nieboer,^64^ József Nyirádi,^2^ Otesile Olubukola,^79^ Matej Oresic,^94^ Fabrizio Ortolano,^27^ Aarno Palotie,^95, 96, 97^ Paul M. Parizel,^98^ Jean-François Payen,^99^ Natascha Perera,^12^ Vincent Perlbarg,^16^ Paolo Persona,^100^ Wilco Peul,^101^ Anna Piippo-Karjalainen,^102^ Matti Pirinen,^95^ Dana Pisica,^64^ Horia Ples,^93^ Suzanne Polinder,^64^ Inigo Pomposo,^29^ Jussi P. Posti,^103^ Louis Puybasset,^104^ Andreea Radoi,^105^ Arminas Ragauskas,^106^ Rahul Raj,^102^ Malinka Rambadagalla,^107^ Isabel Retel Helmrich,^64^ Jonathan Rhodes,^108^ Sylvia Richardson,^109^ Sophie Richter,^47^ Samuli Ripatti,^95^ Saulius Rocka,^106^ Cecilie Roe,^110^ Olav Roise,^111,112^ Jonathan Rosand,^113^ Jeffrey V. Rosenfeld,^114^ Christina Rosenlund,^115^ Guy Rosenthal,^55^ Rolf Rossaint,^77^ Sandra Rossi,^100^ Daniel Rueckert,^61^ Martin Rusnák,^116^ Juan Sahuquillo,^105^ Oliver Sakowitz,^90,117^ Renan Sanchez-Porras,^117^ Janos Sandor,^118^ Nadine Schäfer,^81^ Silke Schmidt,^119^ Herbert Schoechl,^120^ Guus Schoonman,^121^ Rico Frederik Schou,^122^ Elisabeth Schwendenwein,^6^ Charlie Sewalt,^64^ Ranjit D. Singh,^101^ Toril Skandsen,^123,124^ Peter Smielewski,^26^ Abayomi Sorinola,^125^ Emmanuel Stamatakis,^47^ Simon Stanworth,^39^ Robert Stevens,^126^ William Stewart,^127^ Ewout W. Steyerberg,^64,128^ Nino Stocchetti,^129^ Nina Sundström,^130^ Riikka Takala,^131^ Viktória Tamás,^125^ Tomas Tamosuitis,^132^ Mark Steven Taylor,^20^ Aurore Thibaut,^78^ Braden Te Ao,^52^ Olli Tenovuo,^103^ Alice Theadom,^52^ Matt Thomas,^87^ Dick Tibboel,^133^ Marjolein Timmers,^74^ Christos Tolias,^134^ Tony Trapani,^28^ Cristina Maria Tudora,^93^ Andreas Unterberg,^90^ Peter Vajkoczy,^135^ Shirley Vallance,^28^ Egils Valeinis,^60^ Zoltán Vámos,^50^ Mathieu van der Jagt,^136^ Gregory Van der Steen,^43^ Joukje van der Naalt,^71^ Jeroen T.J.M. van Dijck,^101^ Inge A. M. van Erp,^101^ Thomas A. van Essen,^101^ Wim Van Hecke,^137^ Caroline van Heugten,^138^ Dominique Van Praag,^139^ Ernest van Veen,^64^ Thijs Vande Vyvere,^137^ Roel P. J. van Wijk,^101^ Alessia Vargiolu,^32^ Emmanuel Vega,^83^ Kimberley Velt,^64^ Jan Verheyden,^137^ Paul M. Vespa,^140^ Anne Vik,^123,141^ Rimantas Vilcinis,^132^ Victor Volovici,^67^ Nicole von Steinbüchel,^38^ Daphne Voormolen,^64^ Petar Vulekovic,^46^ Kevin K.W. Wang,^142^ Daniel Whitehouse,^47^ Eveline Wiegers,^64^ Guy Williams,^47^ Lindsay Wilson,^69^ Stefan Winzeck,^47^ Stefan Wolf,^143^ Zhihui Yang,^113^ Peter Ylén,^144^ Alexander Younsi,^90^ Frederick A. Zeiler,^47,145^ Veronika Zelinkova,^20^ Agate Ziverte,^60^ Tommaso Zoerle^27^

^1^Department of Physiology and Pharmacology, Section of Perioperative Medicine and Intensive Care, Karolinska Institutet, Stockholm, Sweden

^2^János Szentágothai Research Centre, University of Pécs, Pécs, Hungary

^3^Division of Clinical Neuroscience, Department of Physical Medicine and Rehabilitation, Oslo University Hospital and University of Oslo, Oslo, Norway

^4^Department of Neurosurgery, University Hospital Northern Norway, Tromso, Norway

^5^Department of Physical Medicine and Rehabilitation, University Hospital Northern Norway, Tromso, Norway

^6^Trauma Surgery, Medical University Vienna, Vienna, Austria

^7^Department of Anesthesiology & Intensive Care, University Hospital Nancy, Nancy, France

^8^Raymond Poincare hospital, Assistance Publique –Hopitaux de Paris, Paris, France

^9^Department of Anesthesiology & Intensive Care, S Raffaele University Hospital, Milan, Italy

^10^Department of Neurosurgery, Radboud University Medical Center, Nijmegen, The Netherlands

^11^Department of Neurosurgery, University of Szeged, Szeged, Hungary

^12^International Projects Management, ARTTIC, Munchen, Germany

^13^Department of Neurology, Neurological Intensive Care Unit, Medical University of Innsbruck, Innsbruck, Austria

^14^Department of Neurosurgery & Anesthesia & intensive care medicine, Karolinska University Hospital, Stockholm, Sweden

^15^NIHR Surgical Reconstruction and Microbiology Research Centre, Birmingham, UK

^16^Anesthesie-Réanimation, Assistance Publique – Hopitaux de Paris, Paris, France

^17^Department of Anesthesia & ICU, AOU Città della Salute e della Scienza di Torino - Orthopedic and Trauma Center, Torino, Italy

^18^Department of Neurology, Odense University Hospital, Odense, Denmark

^19^BehaviourWorks Australia, Monash Sustainability Institute, Monash University, Victoria, Australia

^20^Department of Public Health, Faculty of Health Sciences and Social Work, Trnava University, Trnava, Slovakia

^21^Quesgen Systems Inc., Burlingame, California, USA

^22^Australian & New Zealand Intensive Care Research Centre, Department of Epidemiology and Preventive Medicine, School of Public Health and Preventive Medicine, Monash University, Melbourne, Australia

^23^Department of Surgery and Perioperative Science, Umeå University, Umeå, Sweden

^24^Department of Neurosurgery, Medical School, University of Pécs, Hungary and Neurotrauma Research Group, János Szentágothai Research Centre, University of Pécs, Hungary

^25^Department of Medical Psychology, Universitätsklinikum Hamburg-Eppendorf, Hamburg, Germany

^26^Brain Physics Lab, Division of Neurosurgery, Dept of Clinical Neurosciences, University of Cambridge, Addenbrooke's Hospital, Cambridge, UK

^27^Neuro ICU, Fondazione IRCCS Cà Granda Ospedale Maggiore Policlinico, Milan, Italy

^28^ANZIC Research Centre, Monash University, Department of Epidemiology and Preventive Medicine, Melbourne, Victoria, Australia

^29^Department of Neurosurgery, Hospital of Cruces, Bilbao, Spain

^30^NeuroIntensive Care, Niguarda Hospital, Milan, Italy

^31^School of Medicine and Surgery, Università Milano Bicocca, Milano, Italy

^32^NeuroIntensive Care Unit, Department Neuroscience, IRCCS Fondazione San Gerardo dei Tintori, Monza, Italy

^33^Department of Neurosurgery, Medical Faculty RWTH Aachen University, Aachen, Germany

^34^Department of Anesthesiology and Intensive Care Medicine, University Hospital Bonn, Bonn, Germany

^35^Department of Anesthesia & Neurointensive Care, Cambridge University Hospital NHS Foundation Trust, Cambridge, UK

^36^School of Public Health & PM, Monash University and The Alfred Hospital, Melbourne, Victoria, Australia

^37^Radiology/MRI department, MRC Cognition and Brain Sciences Unit, Cambridge, UK

^38^Institute of Medical Psychology and Medical Sociology, Universitätsmedizin Göttingen, Göttingen, Germany

^39^Oxford University Hospitals NHS Trust, Oxford, UK

^40^Intensive Care Unit, CHU Poitiers, Potiers, France

^41^University of Manchester NIHR Biomedical Research Centre, Critical Care Directorate, Salford Royal Hospital NHS Foundation Trust, Salford, UK

^42^Movement Science Group, Faculty of Health and Life Sciences, Oxford Brookes University, Oxford, UK

^43^Department of Neurosurgery, Antwerp University Hospital and University of Antwerp, Edegem, Belgium

^44^Department of Anesthesia & Intensive Care, Maggiore Della Carità Hospital, Novara, Italy

^45^Department of Neurosurgery, University Hospitals Leuven, Leuven, Belgium

^46^Department of Neurosurgery, Clinical centre of Vojvodina, Faculty of Medicine, University of Novi Sad, Novi Sad, Serbia

^47^Division of Anaesthesia, University of Cambridge, Addenbrooke's Hospital, Cambridge, UK

^48^Center for Stroke Research Berlin, Charité – Universitätsmedizin Berlin, corporate member of Freie Universität Berlin, Humboldt-Universität zu Berlin, and Berlin Institute of Health, Berlin, Germany

^49^Intensive Care Unit, CHR Citadelle, Liège, Belgium

^50^Department of Anaesthesiology and Intensive Therapy, University of Pécs, Pécs, Hungary

^51^Departments of Neurology, Clinical Neurophysiology and Neuroanesthesiology, Region Hovedstaden Rigshospitalet, Copenhagen, Denmark

^52^National Institute for Stroke and Applied Neurosciences, Faculty of Health and Environmental Studies, Auckland University of Technology, Auckland, New Zealand

^53^Department of Neurology, Erasmus MC, Rotterdam, the Netherlands

^54^Department of Anesthesiology and Intensive care, University Hospital Northern Norway, Tromso, Norway

^55^Department of Neurosurgery, Hadassah-hebrew University Medical center, Jerusalem, Israel

^56^Fundación Instituto Valenciano de Neurorrehabilitación (FIVAN), Valencia, Spain

^57^Department of Neurosurgery, Shanghai Renji hospital, Shanghai Jiaotong University/school of medicine, Shanghai, China

^58^Karolinska Institutet, INCF International Neuroinformatics Coordinating Facility, Stockholm, Sweden

^59^Emergency Department, CHU, Liège, Belgium

^60^Neurosurgery clinic, Pauls Stradins Clinical University Hospital, Riga, Latvia

^61^Department of Computing, Imperial College London, London, UK

^62^Department of Neurosurgery, Hospital Universitario 12 de Octubre, Madrid, Spain

^63^Department of Anesthesia, Critical Care and Pain Medicine, Medical University of Vienna, Austria

^64^Department of Public Health, Erasmus Medical Center-University Medical Center, Rotterdam, The Netherlands

^65^College of Health and Medicine, Australian National University, Canberra, Australia

^66^Department of Neurosurgery, Neurosciences Centre & JPN Apex trauma centre, All India Institute of Medical Sciences, New Delhi-110029, India

^67^Department of Neurosurgery, Erasmus MC, Rotterdam, the Netherlands

^68^Department of Neurosurgery, Oslo University Hospital, Oslo, Norway

^69^Division of Psychology, University of Stirling, Stirling, UK

^70^Division of Neurosurgery, Department of Clinical Neurosciences, Addenbrooke's Hospital & University of Cambridge, Cambridge, UK

^71^Department of Neurology, University of Groningen, University Medical Center Groningen, Groningen, Netherlands

^72^Neurointensive Care, Sheffield Teaching Hospitals NHS Foundation Trust, Sheffield, UK

^73^Salford Royal Hospital NHS Foundation Trust Acute Research Delivery Team, Salford, UK

^74^Department of Intensive Care and Department of Ethics and Philosophy of Medicine, Erasmus Medical Center, Rotterdam, The Netherlands

^75^Department of Clinical Neuroscience, Neurosurgery, Umeå University, Umeå, Sweden

^76^Hungarian Brain Research Program - Grant No. KTIA_13_NAP-A-II/8, University of Pécs, Pécs, Hungary

^77^Department of Anaesthesiology, University Hospital of Aachen, Aachen, Germany

^78^Cyclotron Research Center , University of Liège, Liège, Belgium

^79^Centre for Urgent and Emergency Care Research (CURE), Health Services Research Section, School of Health and Related Research (ScHARR), University of Sheffield, Sheffield, UK

^80^Emergency Department, Salford Royal Hospital, Salford UK

^81^Institute of Research in Operative Medicine (IFOM), Witten/Herdecke University, Cologne, Germany

^82^VP Global Project Management CNS, ICON, Paris, France

^83^Department of Anesthesiology-Intensive Care, Lille University Hospital, Lille, France

^84^Department of Neurosurgery, Rambam Medical Center, Haifa, Israel

^85^Department of Anesthesiology & Intensive Care, University Hospitals Southhampton NHS Trust, Southhampton, UK

^86^Cologne-Merheim Medical Center (CMMC), Department of Traumatology, Orthopedic Surgery and Sportmedicine, Witten/Herdecke University, Cologne, Germany

^87^Intensive Care Unit, Southmead Hospital, Bristol, Bristol, UK

^88^Department of Neurological Surgery, University of California, San Francisco, California, USA

^89^Department of Anesthesia & Intensive Care,M. Bufalini Hospital, Cesena, Italy

^90^Department of Neurosurgery, University Hospital Heidelberg, Heidelberg, Germany

^91^Department of Neurosurgery, The Walton centre NHS Foundation Trust, Liverpool, UK

^92^Department of Medical Genetics, University of Pécs, Pécs, Hungary

^93^Department of Neurosurgery, Emergency County Hospital Timisoara , Timisoara, Romania

^94^School of Medical Sciences, Örebro University, Örebro, Sweden

^95^Institute for Molecular Medicine Finland, University of Helsinki, Helsinki, Finland

^96^Analytic and Translational Genetics Unit, Department of Medicine; Psychiatric & Neurodevelopmental Genetics Unit, Department of Psychiatry; Department of Neurology, Massachusetts General Hospital, Boston, MA, USA

^97^Program in Medical and Population Genetics; The Stanley Center for Psychiatric Research, The Broad Institute of MIT and Harvard, Cambridge, MA, USA

^98^Department of Radiology, University of Antwerp, Edegem, Belgium

^99^Department of Anesthesiology & Intensive Care, University Hospital of Grenoble, Grenoble, France

^100^Department of Anesthesia & Intensive Care, Azienda Ospedaliera Università di Padova, Padova, Italy

^101^Dept. of Neurosurgery, Leiden University Medical Center, Leiden, The Netherlands and Dept. of Neurosurgery, Medical Center Haaglanden, The Hague, The Netherlands

^102^Department of Neurosurgery, Helsinki University Central Hospital

^103^Division of Clinical Neurosciences, Department of Neurosurgery and Turku Brain Injury Centre, Turku University Hospital and University of Turku, Turku, Finland

^104^Department of Anesthesiology and Critical Care, Pitié -Salpêtrière Teaching Hospital, Assistance Publique, Hôpitaux de Paris and University Pierre et Marie Curie, Paris, France

^105^Neurotraumatology and Neurosurgery Research Unit (UNINN), Vall d'Hebron Research Institute, Barcelona, Spain

^106^Department of Neurosurgery, Kaunas University of technology and Vilnius University, Vilnius, Lithuania

^107^Department of Neurosurgery, Rezekne Hospital, Latvia

^108^Department of Anaesthesia, Critical Care & Pain Medicine NHS Lothian & University of Edinburg, Edinburgh, UK

^109^Director, MRC Biostatistics Unit, Cambridge Institute of Public Health, Cambridge, UK

^110^Department of Physical Medicine and Rehabilitation, Oslo University Hospital/University of Oslo, Oslo, Norway

^111^Division of Orthopedics, Oslo University Hospital, Oslo, Norway

^112^Institue of Clinical Medicine, Faculty of Medicine, University of Oslo, Oslo, Norway

^113^Broad Institute, Cambridge MA Harvard Medical School, Boston MA, Massachusetts General Hospital, Boston MA, USA

^114^National Trauma Research Institute, The Alfred Hospital, Monash University, Melbourne, Victoria, Australia

^115^Department of Neurosurgery, Odense University Hospital, Odense, Denmark

^116^International Neurotrauma Research Organisation, Vienna, Austria

^117^Klinik für Neurochirurgie, Klinikum Ludwigsburg, Ludwigsburg, Germany

^118^Division of Biostatistics and Epidemiology, Department of Preventive Medicine, University of Debrecen, Debrecen, Hungary

^119^Department Health and Prevention, University Greifswald, Greifswald, Germany

^120^Department of Anaesthesiology and Intensive Care, AUVA Trauma Hospital, Salzburg, Austria

^121^Department of Neurology, Elisabeth-TweeSteden Ziekenhuis, Tilburg, the Netherlands

^122^Department of Neuroanesthesia and Neurointensive Care, Odense University Hospital, Odense, Denmark

^123^Department of Neuromedicine and Movement Science, Norwegian University of Science and Technology, NTNU, Trondheim, Norway

^124^Department of Physical Medicine and Rehabilitation, St.Olavs Hospital, Trondheim University Hospital, Trondheim, Norway

^125^Department of Neurosurgery, University of Pécs, Pécs, Hungary

^126^Division of Neuroscience Critical Care, John Hopkins University School of Medicine, Baltimore, USA

^127^Department of Neuropathology, Queen Elizabeth University Hospital and University of Glasgow, Glasgow, UK

^128^Dept. of Department of Biomedical Data Sciences, Leiden University Medical Center, Leiden, The Netherlands

^129^Department of Pathophysiology and Transplantation, Milan University, and Neuroscience ICU, Fondazione IRCCS Cà Granda Ospedale Maggiore Policlinico, Milano, Ital

^130^Department of Radiation Sciences, Biomedical Engineering, Umeå University, Umeå, Sweden

^131^Perioperative Services, Intensive Care Medicine and Pain Management, Turku University Hospital and University of Turku, Turku, Finland

^132^Department of Neurosurgery, Kaunas University of Health Sciences, Kaunas, Lithuania

^133^Intensive Care and Department of Pediatric Surgery, Erasmus Medical Center, Sophia Children's Hospital, Rotterdam, The Netherlands

^134^Department of Neurosurgery, Kings college London, London, UK

^135^Neurologie, Neurochirurgie und Psychiatrie, Charité – Universitätsmedizin Berlin, Berlin, Germany

^136^Department of Intensive Care Adults, Erasmus MC– University Medical Center Rotterdam, Rotterdam, the Netherlands

^137^icoMetrix NV, Leuven, Belgium

^138^Movement Science Group, Faculty of Health and Life Sciences, Oxford Brookes University, Oxford, UK

^139^Psychology Department, Antwerp University Hospital, Edegem, Belgium

^140^Director of Neurocritical Care, University of California, Los Angeles, USA

^141^Department of Neurosurgery, St.Olavs Hospital, Trondheim University Hospital, Trondheim, Norway

^142^Department of Emergency Medicine, University of Florida, Gainesville, Florida, USA

^143^Department of Neurosurgery, Charité– Universitätsmedizin Berlin, corporate member of Freie Universität Berlin, Humboldt-Universität zu Berlin, and Berlin Institute of Health, Berlin, Germany

^144^VTT Technical Research Centre, Tampere, Finland

^145^Section of Neurosurgery, Department of Surgery, Rady Faculty of Health Sciences, University of Manitoba, Winnipeg, MB, Canada.
