# Supplementary material for: Intramural Healthcare Consumption and Costs After Traumatic Brain Injury: A Collaborative European NeuroTrauma Effectiveness Research in Traumatic Brain Injury (CENTER-TBI) Study
Source: J Neurotrauma. 2023 Sep 29;40(19-20):2126–45. doi: 10.1089/neu.2022.0429 (PMC10541942; doi:10.1089/neu.2022.0429)
Supplement: Supplementary file 1 [file SuppMethods.odt]

## Supplementary Figure S1. Proportion of mean total intramural costs per cost- category according country

| Cost category | AT | BE | DE | DK | ES | FI | FR | HU | IT | LT | LV | NL | NO | RO | RS | SE | UK |
| --- | --- | --- | --- | --- | --- | --- | --- | --- | --- | --- | --- | --- | --- | --- | --- | --- | --- |
|  | Prehospital costs | 6% | 5% | 5% | 5% | 3% | 5% | 3% | 4% | 3% | 2% | 6% | 5% | 5% | 6% | 11% | 3% | 4% |
|  | ICU admission | 65% | 49% | 54% | 48% | 50% | 38% | 64% | 60% | 43% | 43% | 31% | 36% | 38% | 55% | 48% | 44% | 49% |
|  | Ward admission | 11% | 14% | 5% | 4% | 17% | 10% | 6% | 3% | 13% | 16% | 36% | 17% | 9% | 28% | 18% | 7% | 14% |
|  | Intracranial surgery | 5% | 6% | 7% | 12% | 5% | 10% | 8% | 13% | 7% | 12% | 11% | 8% | 9% | 5% | 7% | 13% | 9% |
|  | Extracranial surgery | 0% | 4% | 6% | 2% | 5% | 2% | 1% | 2% | 6% | 1% | 2% | 5% | 7% | 0% | 2% | 4% | 2% |
|  | Laboratory costs | 2% | 2% | 2% | 1% | 3% | 2% | 2% | 2% | 2% | 2% | 4% | 2% | 2% | 4% | 3% | 2% | 2% |
|  | Imaging | 1% | 1% | 1% | 0% | 1% | 2% | 1% | 1% | 1% | 1% | 1% | 1% | 1% | 1% | 3% | 1% | 1% |
|  | Bloodproducts | 0% | 0% | 2% | 1% | 1% | 1% | 1% | 2% | 2% | 1% | 0% | 1% | 1% | 0% | 1% | 4% | 1% |
|  | Rehabilitation | 8% | 17% | 17% | 26% | 14% | 29% | 15% | 12% | 23% | 20% | 6% | 24% | 27% | 0% | 5% | 21% | 16% |

In this figure the proportion of the total intramural costs from each cost category are plotted in a histogram for each country separately. The exact percentage for each cost category (including prehospital costs, intensive care unit and ward admission costs, intra- and extracranial surgery costs, laboratory costs, imaging costs, blood products costs and rehabilitation costs) are presented in the table directly below the figure. 

## Supplementary Table S1. Healthcare services including unit prices and corresponding sources 

| Health care unit | Cost category | Value (€) | Reference | Source | Value (€) 2017 | Remark |
| --- | --- | --- | --- | --- | --- | --- |
| Transportation |  |
| Ambulance | Other | 741 | 2019 | NZA prices | 718 | Costs for  immediate transportations |
| 323 | 2019 | NZA prices | 313 | Costs for planned transportations |
| Mobile Intensive Care Unit | Other | 2,322 | 2019 | NZA prices | 2,250 | Costs for planned ICU transportations |
| Mobile Medical Team | Other | 3,424 | 2011 | Multiple Sources | 3,718 | Per unit. See * for calculations |
| Admission |  |
| ER visit | Admission | 259 | 2014 | Guidelines | 264 | Per visit |
| Ward (academic hospital) | Admission | 642 | 2014 | Guidelines | 656 | Costs per day |
| ICU | Admission | 3,186 | 2021 | NZA prices | 3,001 | Costs per day |
| Rehabilitation | Admission | 460 | 2014 | Guidelines | 471 | Costs per day |
| Imaging |  |
| CT Brain | Diagnostics | 144 | 2020 | NZA prices | 137 | Per unit |
| MRI Brain | Diagnostics | 298 | 2020 | NZA prices | 284 | Per unit |
| Angiography | Diagnostics | 478 | 2018 | NZA prices | 471 | Per unit |
| Intracranial surgical interventions |  |
| Aneurysm | Surgery | 6,586 | 2021 | NZA prices | 6,206 | Brain surgery, without admission costs |
| Acute subdural hematoma | Surgery | 5,609 | 2021 | NZA prices | 5,285 | Brain surgery, without admission costs |
| Contusion | Surgery | 5,609 | 2021 | NZA prices | 5,285 | Brain surgery, without admission costs |
| Craniofacial surgery | Surgery | 1,945 | 2020 | NZA prices | 1,855 | Brain surgery, without admission costs |
| CSF- shunt | Surgery | 4,631 | 2021 | NZA prices | 4,363 | Brain surgery, without admission costs |
| Chronic subdural hematoma | Surgery | 4,631 | 2021 | NZA prices | 4,363 | Brain surgery, without admission costs |
| Decompressive craniectomy | Surgery | 5,609 | 2021 | NZA prices | 5,285 | Brain surgery, without admission costs |
| Depressed skull fracture | Surgery | 5,609 | 2021 | NZA prices | 5,285 | Brain surgery, without admission costs |
| Epidural hematoma | Surgery | 5,609 | 2021 | NZA prices | 5,285 | Brain surgery, without admission costs |
| Intracerebral hematoma | Surgery | 5,609 | 2021 | NZA prices | 5,285 | Brain surgery, without admission costs |
| Infection | Surgery | 4,631 | 2021 | NZA prices | 4,363 | Brain surgery, without admission costs |
| Optic nerve decompression | Surgery | 5,609 | 2021 | NZA prices | 5,285 | Brain surgery, without admission costs |
| Fossa posterior surgery | Surgery | 5,609 | 2021 | NZA prices | 5,285 | Brain surgery, without admission costs |
| Skull base fracture | Surgery | 5,609 | 2021 | NZA prices | 5,285 | Brain surgery, without admission costs |
| Ventriculostomy for CSF drainage | Surgery | 4, 631 | 2021 | NZA prices | 4,363 | Brain surgery, without admission costs |
| Debridement-minimal penetrating injury | Surgery | 5,609 | 2021 | NZA prices | 5,285 | Brain surgery, without admission costs |
| Debridement-extensive penetrating injury | Surgery | 5,609 | 2021 | NZA prices | 5,285 | Brain surgery, without admission costs |
| Foreign body removal | Surgery | 5,609 | 2021 | NZA prices | 5,285 | Brain surgery, without admission costs |
| Bone flap replacement | Surgery | 5,609 | 2021 | NZA prices | 5,285 | Brain surgery, without admission costs |
| Cranioplasty | Surgery | 5,609 | 2021 | NZA prices | 5,285 | Brain surgery, without admission costs |
| Decompressive craniectomy-bifrontal | Surgery | 6,586 | 2021 | NZA prices | 6,206 | Brain surgery, without admission costs |
| Decompressive craniectomy - removal boneflap | Surgery | 6,586 | 2021 | NZA prices | 6,206 | Brain surgery, without admission costs |
| Insertion ICP monitor/ Revision of catheter | Surgery | 4,631 | 2021 | NZA prices | 4,363 | Brain surgery, without admission costs |
| Hydrocephalus | Surgery | 5,609 | 2021 | NZA prices | 5,285 | Brain surgery, without admission costs |
| Other | Surgery | 5,609 | 2021 | NZA prices | 5,285 | Brain surgery, without admission costs |
| Extracranial surgical interventions |  |
| Maxillofacial surgery | Surgery | 1,945 | 2020 | NZA prices | 1,855 | Extracranial surgery, without admission costs |
| Extremity fracture lower limb (internal/ external fixation) | Surgery | 3,540 | 2020 | NZA prices | 3,376 | Extracranial surgery, without admission costs |
| Extremity fracture upper limb (internal/ external fixation) | Surgery | 2,795 | 2020 | NZA prices | 2,666 | Extracranial surgery, without admission costs |
| Fasciotomy | Surgery | 2,170 | 2020 | NZA prices | 2,070 | Extracranial surgery, without admission costs |
| Laparotomy | Surgery | 7,196 | 2021 | NZA prices | 6,781 | Extracranial surgery, without admission costs |
| Pelvic fracture (internal/ external fixation) | Surgery | 9,772 | 2021 | NZA prices | 9,208 | Extracranial surgery, without admission costs |
| Spinal stabilization/ (cervical/ thoracic/ lumbar) | Surgery | 11,685 | 2021 | NZA prices | 11,011 | Extracranial surgery, without admission costs |
| Thoracotomy | Surgery | 7,196 | 2021 | NZA prices | 6,781 | Extracranial surgery, without admission  Costs |
| Tracheostomy | Surgery | 2,113 | 2021 | NZA prices | 1,992 | Extracranial surgery, without admission costs |
| Vascular (operative) | Surgery | 7,196 | 2021 | NZA prices | 6,781 | Extracranial surgery, without admission costs |
| Vascular (endovascular) | Surgery | 19,510 | 2020 | NZA prices | 18,607 | Extracranial surgery, without admission costs |
| Wound closure/graft | Surgery | 2,410 | 2020 | NZA prices | 2,298 | Extracranial surgery, without admission costs |
| Removal osteosynthesis material | Surgery | 1,515 | 2017 | NZA prices | 1,515 | Extracranial surgery, without admission costs |
| Heart catheterization | Surgery | 1,560 | 2016 | NZA prices | 1,581 | Extracranial surgery, without admission costs |
| Pacemaker insertion | Surgery | 4,445 | 2012 | NZA prices | 4,708 | Extracranial surgery, without admission costs |
| Gastrostomy | Surgery | 489 | 2021 | NZA prices | 461 | Extracranial surgery, without admission costs |
| Placement of PEG tube | Surgery | 192 | 2021 | NZA prices | 182 | Extracranial surgery, without admission costs |
| Placement of thoracic drain | Surgery | 1,162 | 2013 | NZA prices | 1,201 | Extracranial surgery, without admission costs |
| Eye surgery | Surgery | 1,870 | 2017 | NZA prices | 1,870 | Extracranial surgery, without admission costs |
| Ear surgery | Surgery | 765 | 2017 | NZA prices | 765 | Extracranial surgery, without admission costs |
| Other unspecified surgery | Surgery | 2,215 | 2020 | NZA prices | 2,113 | Extracranial surgery, without admission costs |
| Laboratory |  |
| Hematology and blood chemistry** | Laboratory | 67 | 2021 | [Tarieven (star-shl.nl)](https://www.star-shl.nl/patient/tarieven/) | 63 | Per unit |
| Packed red blood cell concentrates | Laboratory | 216 | 2014 | Guidelines | 221 | Per unit, 280 ml |
| Fresh whole blood | Laboratory | 216 | 2014 | Guidelines | 221 | Per unit, 280 ml |
| Fresh frozen plasma | Laboratory | 163 | 2021 | Medicijnkosten.nl | 154 | Per unit, 200 ml |
| Freeze dried plasma | Laboratory | 163 | 2021 | Medicijnkosten.nl | 154 | Per unit, 200 ml |
| Platelet concentrates | Laboratory | 522 | 2014 | Guidelines | 534 | Per unit, 330 ml |
| Prothrombin complex | Laboratory | 460 | 2021 | Medicijnkosten.nl | 433 | Per unit, 500IE |
| Fibrinogen concentrate | Laboratory | 2,581 | 2021 | Medicijnkosten.nl | 2,432 | Per unit , 1gr |
| Albumine | Laboratory | 286 | 2021 | Medicijnkosten.nl | 269 | Per unit, 40mg/ml (100ml) |
| Recombinant factor FVIIa | Laboratory | 820 | 2021 | Medicijnkosten.nl | 773 | Per unit, 1mg |
| Tranexamic acid | Laboratory | 17 | 2021 | Medicijnkosten.nl | 16 | Per unit, 100mg |
| Cryoprecipate | Laboratory | 710 | 2021 | Medicijnkosten.nl | 669 | Per unit, 10.000E (50mg) |
| Desmopressin | Laboratory | 14 | 2021 | Medicijnkosten.nl | 13 | Per unit, 4mcg/ml (ampul 1ml) |
| Factor XIII | Laboratory | 1,030 | 2021 | Medicijnkosten.nl | 971 | Per unit, 1000IE |
| Calcium | Laboratory | 7 | 2021 | Medicijnkosten.nl | 6 | Per unit, 100mg/ml (ampul 10ml) |
| Vitamin K | Laboratory | 4 | 2021 | Medicijnkosten.nl | 3 | Per unit, 10mg/g |

Caption: 

Supplementary table S1 shows a detailed overview of all used unit costs and corresponding sources. Using the national general consumer price index of the Netherlands all costs were corrected to Euro 2017, available from the Central Bureau of statistics: [https://opendata.cbs.nl/statline/#/CBS/nl/dataset/70936ned/table?ts=1551859688904](#/CBS/nl/dataset/70936ned/table?ts=1551859688904) 

Legend: 

*Own calculation: National total costs for 2011: 21.3 million EUR (source: [https://www.recht.nl/rechtspraak/uitspraak/?ecli=ECLI:NL:CBB:2015:428](https://eur01.safelinks.protection.outlook.com/?url=https%3A%2F%2Fwww.recht.nl%2Frechtspraak%2Fuitspraak%2F%3Fecli%3DECLI%3ANL%3ACBB%3A2015%3A428&data=04%7C01%7Cz.kaplan%40erasmusmc.nl%7C4e5c45a21cc44a1d231408d92109dc86%7C526638ba6af34b0fa532a1a511f4ac80%7C0%7C0%7C637577148494248270%7CUnknown%7CTWFpbGZsb3d8eyJWIjoiMC4wLjAwMDAiLCJQIjoiV2luMzIiLCJBTiI6Ik1haWwiLCJXVCI6Mn0%3D%7C1000&sdata=uonyRqU6khjlIa%2Bv%2FRHehnApJaGk0qV81Ij%2Fxpox2gw%3D&reserved=0); accessed 06-09-2019. National total use: 6220 units (https://www.ambulancezorg.nl/static/upload/raw/1aa59bb7-a8e0-48e5-897e-06f9703a7e3d/ambulances-in-zicht-2015.pdf; accessed 06-09-2019). Unit price =€3,424 (21,300,000/6.223).
**Due to the major variation in laboratory studies only the most commonly performed studies were counted. Prices are calculated based on the NZA prices of individual studies.  

## Supplementary Table S2. National general consumer price index, the Netherlands

| Year | Inflation % | Absolute inflation | Calculation factor |
| --- | --- | --- | --- |
| 2017 | 1.40% | 1.014 | 0.986 |
| 2018 | 1.70% | 1.017 | 0.983 |
| 2019 | 1.60% | 1.016 | 0.984 |
| 2020 | 1.20% | 1.012 | 0.988 |

## Supplementary Table S3. Purchasing power parity (PPP) for the general domestic product (GDP)

| Country | PPP for GDP National currency per US Dollar- 2017 | PPP for GDP in US Dollar- 2017* |
| --- | --- | --- |
| Netherlands | 0.782 | 1.278772379 |
| Belgium | 0.776 | 1.288659794 |
| France | 0.77 | 1.298701299 |
| Germany | 0.745 | 1.342281879 |
| Spain | 0.631 | 1.584786054 |
| Italy | 0.69 | 1.449275362 |
| United Kingdom | 0.685 | 1.459854015 |
| Serbia | 40.691 | 0.024575459 |
| Romania | 1.614 | 0.619578686 |
| Hungary | 136.037 | 0.007350941 |
| Austria | 0.775 | 1.290322581 |
| Lithuania | 0.443 | 2.257336343 |
| Latvia | 0.485 | 2.06185567 |
| Denmark | 6.872 | 0.145518044 |
| Finland | 0.864 | 1.157407407 |
| Sweden | 8.852 | 0.112968821 |

* PPP for GDP in US dollar is calculated by 1/ PPP for GDP in national currency. For example: The Netherlands has a PPP for GDP of 0.782 in their national currency per US dollar, which is 1.278772379 (1/0.782) for PPP for GDP in US dollars. 

PPP-GDP correction available from: [Purchasing Power Parities for GDP and related indicators (oecd.org)](https://stats.oecd.org/Index.aspx?DataSetCode=PPPGDP)

## Supplementary Table S4. Proportion of total costs spend on different categories according to severity of TBI

| Cost category | TBI Severity | p - value |
| --- | --- | --- |
| Mild | Moderate | Severe |  | Total |
| Mean | sd. | (%) | Mean | sd. | (%) | Mean | sd. | (%) | Mean | sd. | (%) |  |
| Total costs | 14800 | 29800 | 100.0% | 54700 | 55000 | 100.0% | 79000 | 74800 | 100.0% | 33200 | 54100 | 100.0% | <0.001 |
| Prehospital costs | 1000 | 1200 | 7.0% | 1700 | 1600 | 3.2% | 2200 | 1800 | 2.8% | 1400 | 1500 | 4.1% | <0.001 |
| ICU admission | 5300 | 16500 | 35.6% | 26600 | 33100 | 48.7% | 41700 | 48800 | 52.8% | 15600 | 32800 | 47.0% | <0.001 |
| Ward admission | 2900 | 6300 | 19.6% | 6800 | 9600 | 12.4% | 6800 | 12700 | 8.6% | 4100 | 8700 | 12.5% | <0.001 |
| Intracranial surgery | 900 | 3100 | 6.3% | 4800 | 6200 | 8.9% | 7100 | 8300 | 9.0% | 2700 | 5800 | 8.2% | <0.001 |
| Extracranial surgery | 900 | 3400 | 6.0% | 1800 | 5000 | 3.4% | 2200 | 5100 | 2.8% | 1300 | 4100 | 3.8% | <0.001 |
| Laboratory costs | 400 | 800 | 2.6% | 1200 | 1300 | 2.2% | 1500 | 1700 | 1.9% | 700 | 1200 | 2.2% | <0.001 |
| Imaging | 300 | 200 | 1.7% | 400 | 300 | 0.8% | 500 | 400 | 0.6% | 300 | 300 | 1.0% | <0.001 |
| Bloodproducts | 100 | 800 | 0.8% | 600 | 1600 | 1.2% | 1100 | 4900 | 1.4% | 400 | 2500 | 1.2% | <0.001 |
| Rehabilitation | 2800 | 10400 | 18.6% | 10400 | 19300 | 18.9% | 15600 | 25200 | 19.8% | 6400 | 16800 | 19.3% | <0.001 |

## Supplementary Table S5. Mean intramural costs for each cost category according to baseline characteristics

| Patient characteristics | Cost category |
| --- | --- |
| Total costs | Prehospital | Intracranial | Extracranial | Hospitalization | ICU (LOS. days) | Ward (LOS. days) | Laboratory | Imaging | Bloodproducts | Rehabilitation costs | Rehabiliation (LOS. days) |
| Mean | sd. | Mean | sd. | Mean | sd. | Mean | sd. | Mean | sd. | Mean | sd. | Mean | sd. | Mean | sd. | Mean | sd. | Mean | sd. | Mean | sd. | Mean | sd. |
| Sex |  |  |  |  |  |  |  |  |  |  |  |  |  |  |  |  |  |  |  |  |  |  |  |  |
| Male | 37200 | 58500 | 1500 | 1500 | 3000 | 6200 | 1400 | 4300 | 22800 | 39500 | 5.9 | 11.3 | 6.8 | 13.7 | 800 | 1300 | 300 | 300 | 500 | 2900 | 7000 | 18100 | 14.6 | 37.3 |
| Female | 25000 | 42500 | 1200 | 1300 | 2000 | 4600 | 1000 | 3400 | 14400 | 26600 | 3.5 | 7.6 | 5.4 | 11.5 | 600 | 1000 | 300 | 200 | 300 | 1200 | 5300 | 13900 | 11.1 | 29.0 |
| Age |  |  |  |  |  |  |  |  |  |  |  |  |  |  |  |  |  |  |  |  |  |  |  |  |
| 16-25 years | 36100 | 59300 | 1500 | 1600 | 3100 | 6500 | 1500 | 4700 | 21300 | 36000 | 5.6 | 10.4 | 5.7 | 12.7 | 700 | 1200 | 300 | 300 | 500 | 1900 | 7100 | 18900 | 14.5 | 38.0 |
| 26-40 years | 37200 | 66500 | 1300 | 1400 | 3300 | 7300 | 1700 | 5000 | 22800 | 48000 | 6.0 | 13.2 | 6.3 | 12.7 | 800 | 1300 | 300 | 300 | 600 | 5100 | 6500 | 16200 | 14.2 | 35.8 |
| 41-64 years | 34400 | 53700 | 1400 | 1500 | 2800 | 5500 | 1300 | 3900 | 20700 | 34600 | 5.2 | 10.0 | 6.6 | 13.2 | 800 | 1200 | 300 | 300 | 300 | 1300 | 6700 | 18500 | 13.8 | 36.8 |
| ≥65 years | 27300 | 40000 | 1200 | 1300 | 2000 | 4200 | 700 | 3000 | 16600 | 27500 | 4.1 | 8.4 | 6.4 | 13.2 | 700 | 1100 | 300 | 200 | 300 | 1100 | 5500 | 13200 | 11.9 | 28.8 |
| Medical History |  |  |  |  |  |  |  |  |  |  |  |  |  |  |  |  |  |  |  |  |  |  |  |  |
| Healthy patient | 32800 | 52100 | 1400 | 1500 | 2700 | 5600 | 1500 | 4400 | 19500 | 32800 | 5.2 | 9.9 | 5.8 | 12.0 | 700 | 1100 | 300 | 300 | 400 | 3200 | 6300 | 16700 | 13.3 | 34.6 |
| Mild systemic disease | 33700 | 58300 | 1300 | 1400 | 2700 | 6100 | 1100 | 3800 | 20400 | 40600 | 5.0 | 11.0 | 6.7 | 12.7 | 800 | 1300 | 300 | 300 | 300 | 1300 | 6700 | 17500 | 13.8 | 36.0 |
| Severe systemic disease | 34100 | 51100 | 1300 | 1300 | 2700 | 5200 | 700 | 2700 | 21400 | 36700 | 5.1 | 10.3 | 7.7 | 17.9 | 800 | 1400 | 300 | 300 | 400 | 1200 | 6500 | 15300 | 13.4 | 32.1 |
| Cause of injury |  |  |  |  |  |  |  |  |  |  |  |  |  |  |  |  |  |  |  |  |  |  |  |  |
| Road traffic accident | 39800 | 55900 | 1600 | 1600 | 2900 | 5700 | 2100 | 5300 | 23600 | 35700 | 6.3 | 10.8 | 6.8 | 12.8 | 800 | 1200 | 300 | 300 | 500 | 1700 | 7900 | 18400 | 16.8 | 38.4 |
| Fall | 28600 | 52600 | 1200 | 1300 | 2500 | 5700 | 600 | 2500 | 17600 | 36900 | 4.4 | 10.3 | 5.8 | 12.2 | 600 | 1200 | 300 | 300 | 200 | 1000 | 5500 | 15700 | 11.5 | 32.8 |
| Violence | 26000 | 48500 | 1100 | 1400 | 3300 | 6200 | 300 | 1500 | 15400 | 29600 | 3.4 | 7.3 | 6.1 | 16.7 | 600 | 1200 | 300 | 400 | 700 | 8700 | 4300 | 12800 | 8.9 | 26.4 |
| Self-harm | 70300 | 66900 | 1700 | 1500 | 4500 | 5800 | 6500 | 10200 | 43500 | 45200 | 10.9 | 13.2 | 16.9 | 27.9 | 1700 | 2200 | 400 | 200 | 2100 | 3100 | 10000 | 16500 | 22.5 | 37.0 |
| Other | 29300 | 50700 | 1300 | 1400 | 2400 | 5900 | 1000 | 3400 | 17700 | 32100 | 4.3 | 8.8 | 5.8 | 11.9 | 700 | 1200 | 300 | 200 | 300 | 1500 | 5600 | 17400 | 11.1 | 32.0 |
| TBI severity |  |  |  |  |  |  |  |  |  |  |  |  |  |  |  |  |  |  |  |  |  |  |  |  |
| Mild | 14800 | 29800 | 1000 | 1200 | 900 | 3100 | 900 | 3400 | 8400 | 19100 | 1.8 | 5.5 | 4.5 | 9.5 | 400 | 800 | 300 | 200 | 100 | 800 | 2800 | 10400 | 5.8 | 21.5 |
| Moderate | 54700 | 55000 | 1700 | 1600 | 4800 | 6200 | 1800 | 5000 | 33700 | 36200 | 8.9 | 10.9 | 10.1 | 13.5 | 1200 | 1300 | 400 | 300 | 600 | 1600 | 10400 | 19300 | 22.1 | 41.0 |
| Severe | 79000 | 74800 | 2200 | 1800 | 7100 | 8300 | 2200 | 5100 | 48800 | 52200 | 13.5 | 14.7 | 10.3 | 19.2 | 1500 | 1700 | 500 | 400 | 1100 | 4900 | 15600 | 25200 | 32.6 | 51.7 |
| Brain AIS |  |  |  |  |  |  |  |  |  |  |  |  |  |  |  |  |  |  |  |  |  |  |  |  |
| Minor | 4900 | 11600 | 800 | 900 | 100 | 700 | 700 | 2800 | 2300 | 6900 | 0.3 | 1.7 | 1.9 | 4.6 | 100 | 300 | 200 | 100 | 0 | 500 | 700 | 4800 | 1.4 | 9.8 |
| Moderate | 7700 | 18400 | 800 | 900 | 200 | 900 | 800 | 2900 | 4100 | 12300 | 0.7 | 3.3 | 2.8 | 11.7 | 200 | 800 | 200 | 100 | 100 | 500 | 1300 | 6800 | 2.9 | 14.6 |
| Serious | 12400 | 22900 | 1000 | 1000 | 400 | 1900 | 800 | 3000 | 6600 | 14200 | 1.0 | 3.8 | 5.5 | 9.2 | 400 | 700 | 300 | 200 | 100 | 700 | 2800 | 10700 | 6.1 | 21.9 |
| Severe | 43600 | 48300 | 1700 | 1600 | 2900 | 4700 | 2200 | 5900 | 26700 | 30700 | 7.2 | 9.6 | 8.9 | 13.1 | 1000 | 1100 | 400 | 200 | 500 | 1700 | 8300 | 17100 | 18.1 | 37.6 |
| Critical | 87100 | 72800 | 2300 | 1800 | 8700 | 8300 | 2000 | 4800 | 53900 | 51000 | 14.8 | 14.5 | 11.2 | 19.1 | 1700 | 1700 | 500 | 400 | 1100 | 4800 | 17000 | 25700 | 34.9 | 52.0 |
| Unsurvivable | 18800 | 49900 | 1800 | 1500 | 2700 | 5500 | 700 | 2500 | 11900 | 43200 | 3.4 | 11.4 | 0.8 | 3.5 | 300 | 1000 | 200 | 200 | 900 | 1900 | 300 | 2100 | 0.5 | 3.6 |
| ISS |  |  |  |  |  |  |  |  |  |  |  |  |  |  |  |  |  |  |  |  |  |  |  |  |
| Minor (≤16) | 8700 | 20100 | 800 | 900 | 500 | 2200 | 200 | 1300 | 4700 | 12400 | 0.8 | 3.5 | 3.3 | 8.5 | 200 | 600 | 200 | 200 | 0 | 300 | 1900 | 9000 | 4.1 | 18.8 |
| Major (16-25) | 42200 | 63300 | 1600 | 1400 | 4400 | 7700 | 800 | 2700 | 25700 | 45500 | 6.4 | 12.6 | 7.9 | 12.6 | 900 | 1400 | 400 | 300 | 300 | 1200 | 8200 | 19000 | 16.9 | 38.0 |
| Critically injured (>25) | 69000 | 64400 | 2100 | 1800 | 5300 | 6900 | 3400 | 6500 | 42400 | 42500 | 11.5 | 12.6 | 10.5 | 17.6 | 1400 | 1500 | 400 | 300 | 1100 | 4400 | 13000 | 22300 | 27.2 | 46.7 |
| Baseline pupillary reaction |  |  |  |  |  |  |  |  |  |  |  |  |  |  |  |  |  |  |  |  |  |  |  |  |
| Both reacting | 29700 | 51000 | 1300 | 1400 | 2200 | 5300 | 1200 | 4100 | 17800 | 33800 | 4.4 | 9.5 | 6.2 | 12.8 | 700 | 1200 | 300 | 300 | 300 | 2600 | 5900 | 16100 | 12.4 | 33.4 |
| One reacting | 68200 | 65600 | 1900 | 1700 | 6800 | 7200 | 1700 | 3600 | 40800 | 42900 | 11.0 | 12.1 | 9.1 | 14.7 | 1300 | 1400 | 400 | 300 | 800 | 1600 | 14500 | 24400 | 30.0 | 49.8 |
| Non-reacting | 58900 | 69800 | 2100 | 1800 | 6700 | 7600 | 1600 | 3800 | 37100 | 49400 | 10.6 | 15.2 | 6.3 | 14.5 | 1100 | 1500 | 400 | 400 | 1200 | 2300 | 8700 | 19400 | 18.0 | 39.9 |
| CT abnormalities |  |  |  |  |  |  |  |  |  |  |  |  |  |  |  |  |  |  |  |  |  |  |  |  |
| Any CT abnormality |  |  |  |  |  |  |  |  |  |  |  |  |  |  |  |  |  |  |  |  |  |  |  |  |
| Absent | 16600 | 45200 | 1000 | 1200 | 1100 | 4200 | 900 | 3700 | 9700 | 32700 | 2.3 | 9.1 | 3.7 | 11.0 | 400 | 1000 | 200 | 200 | 100 | 900 | 3100 | 12800 | 6.7 | 26.1 |
| Present | 46900 | 56900 | 1600 | 1600 | 4000 | 6500 | 1600 | 4300 | 28600 | 36400 | 7.4 | 10.7 | 8.5 | 14.1 | 1000 | 1300 | 400 | 300 | 600 | 3300 | 9100 | 19200 | 19.1 | 39.7 |
| Cisternal compression | 70000 | 68000 | 1900 | 1600 | 8400 | 8100 | 1100 | 3200 | 42000 | 43100 | 11.4 | 12.2 | 9.3 | 16.2 | 1300 | 1500 | 500 | 400 | 1200 | 2400 | 13600 | 25100 | 28.2 | 51.6 |
| Midline shift | 64400 | 63600 | 1800 | 1600 | 8700 | 8300 | 700 | 2300 | 37600 | 40100 | 10.0 | 11.4 | 9.1 | 15.3 | 1200 | 1400 | 500 | 400 | 1000 | 2100 | 12900 | 23700 | 27.0 | 49.3 |
| Subarachnoid heamorrhage | 52600 | 59300 | 1700 | 1600 | 4600 | 7000 | 1600 | 4200 | 32200 | 37900 | 8.4 | 11.0 | 9.3 | 15.5 | 1100 | 1400 | 400 | 300 | 600 | 1800 | 10400 | 20600 | 21.6 | 42.5 |
| Epidural hematoma | 53300 | 61600 | 1700 | 1600 | 5700 | 6900 | 1400 | 4000 | 33000 | 39600 | 8.6 | 11.7 | 9.5 | 16.8 | 1200 | 1400 | 500 | 300 | 600 | 1800 | 9400 | 20300 | 19.8 | 43.1 |
| Acute subdural hematoma | 54200 | 61200 | 1700 | 1600 | 5500 | 7600 | 1400 | 4300 | 33000 | 38800 | 8.6 | 11.0 | 9.1 | 15.5 | 1100 | 1400 | 400 | 300 | 700 | 1900 | 10500 | 20800 | 21.8 | 42.8 |
| Diffuse axonal injury | 62300 | 65200 | 2200 | 1900 | 4400 | 6100 | 2200 | 5400 | 37700 | 41500 | 10.2 | 12.2 | 9.1 | 17.0 | 1200 | 1400 | 400 | 300 | 1000 | 6800 | 13100 | 23300 | 26.7 | 46.8 |
| Contusion | 57500 | 62100 | 1800 | 1700 | 5300 | 7400 | 1300 | 3800 | 35700 | 40200 | 9.5 | 11.7 | 9.4 | 15.7 | 1200 | 1400 | 500 | 300 | 700 | 4200 | 11100 | 21200 | 23.0 | 43.7 |
| Inhospital mortality |  |  |  |  |  |  |  |  |  |  |  |  |  |  |  |  |  |  |  |  |  |  |  |  |
| No | 33600 | 55400 | 1300 | 1400 | 2500 | 5700 | 1300 | 4100 | 20100 | 36400 | 5.1 | 10.4 | 6.8 | 13.4 | 800 | 1200 | 300 | 300 | 300 | 2500 | 6900 | 17400 | 14.6 | 36.0 |
| Yes | 28600 | 35000 | 1800 | 1700 | 4800 | 5600 | 1000 | 3200 | 18900 | 30500 | 5.9 | 9.2 | 0.6 | 3.1 | 400 | 700 | 300 | 300 | 1100 | 2400 | 100 | 1700 | - | - |
| GOSE-6 months disability |  |  |  |  |  |  |  |  |  |  |  |  |  |  |  |  |  |  |  |  |  |  |  |  |
| 1 | 38900 | 60800 | 1800 | 1700 | 5100 | 7200 | 1100 | 3500 | 26600 | 51300 | 7.5 | 14.2 | 4.3 | 11.7 | 800 | 1300 | 400 | 300 | 900 | 2100 | 2300 | 8200 | 5.1 | 18.1 |
| 2_3 | 107500 | 80400 | 1900 | 1600 | 8100 | 9300 | 3000 | 6900 | 61500 | 48500 | 16.3 | 14.3 | 17.8 | 27.5 | 2200 | 2000 | 500 | 400 | 1100 | 2300 | 29300 | 34600 | 61.3 | 70.8 |
| 4 | 68400 | 68700 | 1900 | 1700 | 4900 | 7700 | 3200 | 7300 | 41800 | 43000 | 10.7 | 12.1 | 12.3 | 18.2 | 1500 | 1700 | 400 | 400 | 700 | 1500 | 14000 | 22000 | 28.8 | 44.9 |
| 5 | 53100 | 57800 | 1800 | 1700 | 4400 | 6400 | 2000 | 4800 | 31700 | 39700 | 8.2 | 12.3 | 9.4 | 11.1 | 1100 | 1200 | 400 | 300 | 600 | 1800 | 11200 | 17000 | 23.2 | 35.6 |
| 6 | 36300 | 46100 | 1500 | 1500 | 2300 | 4800 | 1900 | 4500 | 21300 | 30400 | 5.4 | 9.0 | 7.2 | 9.6 | 800 | 1000 | 300 | 200 | 600 | 6600 | 7500 | 14500 | 15.9 | 30.8 |
| 7 | 17500 | 28900 | 1100 | 1300 | 1300 | 3700 | 800 | 2900 | 10400 | 19300 | 2.4 | 5.9 | 4.7 | 6.7 | 400 | 600 | 300 | 200 | 100 | 700 | 3000 | 8700 | 6.3 | 18.5 |
| 8 | 11000 | 23800 | 1000 | 1100 | 700 | 2600 | 500 | 2300 | 6600 | 16600 | 1.4 | 4.8 | 3.3 | 8.1 | 300 | 600 | 200 | 200 | 100 | 600 | 1600 | 8000 | 3.3 | 15.8 |

## Supplementary Table S6. Mean standardized intramural costs of traumatic brain injury according to trauma severity 

| Patient characteristics | TBI severity | p - value |
| --- | --- | --- |
| Mild | Moderate | Severe | Total |
| Mean (€) | sd. | Mean (€) | sd. | Mean (€) | sd. | Mean (€) | sd. |  |
| Total | 14800 | 29800 | 54700 | 55000 | 79000 | 74800 | 33200 | 54100 | <0.001 |
| Sex |  |  |  |  |  |  |  |  |  |
| Male | 16400 | 32300 | 58000 | 59000 | 82400 | 78200 | 37200 | 58500 | <0.001 |
| Female | 11900 | 24400 | 47400 | 44300 | 68700 | 62600 | 25000 | 42500 | <0.001 |
| Age |  |  |  |  |  |  |  |  |  |
| 16-25 years | 10900 | 26200 | 58600 | 76600 | 87700 | 71800 | 36100 | 59300 | <0.001 |
| 26-40 years | 14000 | 32600 | 58300 | 55300 | 91800 | 96300 | 37200 | 66500 | <0.001 |
| 41-64 years | 14400 | 28300 | 59900 | 55900 | 83700 | 71500 | 34400 | 53700 | <0.001 |
| ≥65 years | 17900 | 31500 | 45300 | 39500 | 52400 | 52400 | 27300 | 40000 | <0.001 |
| Medical History |  |  |  |  |  |  |  |  |  |
| Healthy patient | 12900 | 27100 | 54500 | 51300 | 81200 | 68200 | 32800 | 52100 | <0.001 |
| Mild systemic disease | 16300 | 31300 | 53500 | 57300 | 79500 | 88500 | 33700 | 58300 | <0.001 |
| Severe systemic disease | 19900 | 36500 | 58700 | 62900 | 65300 | 63100 | 34100 | 51100 | <0.001 |
| Cause of injury |  |  |  |  |  |  |  |  |  |
| Road traffic accident | 16600 | 30900 | 56700 | 52400 | 83300 | 68600 | 39800 | 55900 | <0.001 |
| Fall | 14400 | 30000 | 53500 | 57100 | 71500 | 82200 | 28600 | 52600 | <0.001 |
| Violence | 9400 | 21400 | 52600 | 54600 | 99200 | 72100 | 26000 | 48500 | <0.001 |
| Self-harm | 42600 | 60400 | 104000 | 64900 | 76100 | 67100 | 70300 | 66900 | 0.037 |
| Other | 12100 | 25300 | 43200 | 50100 | 81400 | 74000 | 29300 | 50700 | <0.001 |
| Brain AIS |  |  |  |  |  |  |  |  |  |
| Minor | 4600 | 11400 | 13000 | 11700 | 16300 | 15400 | 4900 | 11600 | <0.001 |
| Moderate | 6000 | 13500 | 5200 | 3800 | 47500 | 50300 | 7700 | 18400 | <0.001 |
| Serious | 11200 | 20900 | 21600 | 27000 | 35700 | 46000 | 12400 | 22900 | <0.001 |
| Severe | 31700 | 39200 | 49800 | 50600 | 64900 | 55800 | 43600 | 48300 | <0.001 |
| Critical | 72200 | 58000 | 73900 | 58700 | 94100 | 78200 | 87100 | 72800 | <0.001 |
| Unsurvivable | 29200 | 33800 | 5000 | 3300 | 19300 | 53200 | 18800 | 49900 | 0.080 |
| ISS |  |  |  |  |  |  |  |  |  |
| Minor (≤16) | 6600 | 14800 | 28100 | 39000 | 39200 | 46400 | 8700 | 20100 | <0.001 |
| Major (16-25) | 22100 | 35200 | 57600 | 51500 | 81500 | 91700 | 42200 | 63300 | <0.001 |
| Critically injured (>25) | 46000 | 48600 | 67900 | 59400 | 82700 | 69700 | 69000 | 64400 | <0.001 |
| Baseline pupillary reaction |  |  |  |  |  |  |  |  |  |
| Both reacting | 14400 | 29200 | 54100 | 55900 | 82500 | 75500 | 29700 | 51000 | <0.001 |
| One reacting | 24400 | 34800 | 64100 | 41300 | 90700 | 69800 | 68200 | 65600 | <0.001 |
| Non-reacting | 32500 | 54200 | 56500 | 52600 | 63500 | 72800 | 58900 | 69800 | <0.001 |
| CT abnormalities |  |  |  |  |  |  |  |  |  |
| Any CT abnormality |  |  |  |  |  |  |  |  |  |
| Absent | 8300 | 22000 | 46100 | 62800 | 75500 | 102200 | 16600 | 45200 | <0.001 |
| Present | 23400 | 36000 | 57200 | 52400 | 79800 | 66700 | 46900 | 56900 | <0.001 |
| Cisternal compression | 54200 | 61700 | 58100 | 51500 | 78500 | 72200 | 70000 | 68000 | <0.001 |
| Midline shift | 47400 | 48800 | 53800 | 48100 | 75400 | 71200 | 64400 | 63600 | 0.003 |
| Subarachnoid      heamorrhage | 27900 | 40300 | 59200 | 54100 | 80800 | 67300 | 52600 | 59300 | <0.001 |
| Epidural hematoma | 30800 | 46700 | 49200 | 48900 | 91200 | 70100 | 53300 | 61600 | <0.001 |
| Acute subdural      hematoma | 27700 | 38100 | 63200 | 56400 | 80100 | 70900 | 54200 | 61200 | <0.001 |
| Diffuse axonal injury | 22100 | 38100 | 66300 | 56700 | 94000 | 66800 | 62300 | 65200 | <0.001 |
| Contusion | 29700 | 41000 | 62900 | 55500 | 87500 | 69900 | 57500 | 62100 | <0.001 |
| Inhospital mortality |  |  |  |  |  |  |  |  |  |
| No | 14600 | 29800 | 58400 | 56700 | 93000 | 76900 | 33600 | 55400 | <0.001 |
| Yes | 23600 | 27200 | 26300 | 25100 | 30400 | 38300 | 28600 | 35000 | 0.069 |
| GOSE-6 months disability |  |  |  |  |  |  |  |  |  |
| 1 | 23900 | 28400 | 42400 | 44200 | 44900 | 73300 | 38900 | 60800 | <0.001 |
| 2_3 | 66600 | 72900 | 108000 | 83300 | 135100 | 72800 | 107500 | 80400 | <0.001 |
| 4 | 34700 | 51600 | 77400 | 67100 | 114000 | 64400 | 68400 | 68700 | <0.001 |
| 5 | 26600 | 36200 | 57400 | 42600 | 94600 | 66600 | 53100 | 57800 | <0.001 |
| 6 | 18900 | 25600 | 54100 | 45900 | 78200 | 60100 | 36300 | 46100 | <0.001 |
| 7 | 10000 | 17000 | 40100 | 35900 | 54800 | 46400 | 17500 | 28900 | <0.001 |
| 8 | 7000 | 14900 | 36200 | 39200 | 50800 | 51300 | 11000 | 23800 | <0.001 |

## Supplementary Table S7. Baseline characteristics according to country of injury and treatment. 

| Patient characteristics | Country |  |
| --- | --- | --- |
| AT | BE | DE | DK | ES | FI | FR | HU | IT | LT | LV | NL | NO | RO | RS | SE | UK | p - value |
| No. | (%) | No. | (%) | No. | (%) | No. | (%) | No. | (%) | No. | (%) | No. | (%) | No. | (%) | No. | (%) | No. | (%) | No. | (%) | No. | (%) | No. | (%) | No. | (%) | No. | (%) | No. | (%) | No. | (%) |  |
| Total | 109 |  | 298 |  | 183 |  | 15 |  | 410 |  | 367 |  | 114 |  | 43 |  | 540 |  | 45 |  | 105 |  | 962 |  | 366 |  | 17 |  | 63 |  | 134 |  | 578 |  |  |
| Sex |  |  |  |  |  |  |  |  |  |  |  |  |  |  |  |  |  |  |  |  |  |  |  |  |  |  |  |  |  |  |  |  |  |  |  |
| Male | 75 | 69% | 210 | 70% | 126 | 69% | 11 | 73% | 284 | 69% | 240 | 65% | 91 | 80% | 25 | 58% | 385 | 71% | 27 | 60% | 72 | 69% | 598 | 62% | 245 | 67% | 13 | 76% | 43 | 68% | 81 | 60% | 400 | 69% | 0.006 |
| Female | 34 | 31% | 88 | 30% | 57 | 31% | 4 | 27% | 126 | 31% | 127 | 35% | 23 | 20% | 18 | 42% | 155 | 29% | 18 | 40% | 33 | 31% | 364 | 38% | 121 | 33% | 4 | 24% | 20 | 32% | 53 | 40% | 178 | 31% |  |
| Age |  |  |  |  |  |  |  |  |  |  |  |  |  |  |  |  |  |  |  |  |  |  |  |  |  |  |  |  |  |  |  |  |  |  |  |
| Median [IQR], years | 63 [52, 75] | 49 [34, 62] | 55 [30, 72] | 41 [33, 67] | 45 [31, 62] | 49 [29, 64] | 35 [23, 55] | 46 [32, 70] | 55 [33, 71] | 49 [36, 63] | 43 [31, 55] | 59 [39, 71] | 46 [25, 63] | 57 [40, 66] | 42 [27, 61] | 51 [33, 63] | 49 [32, 64] | <0.001 |
| ≥65 | 52 | 48% | 63 | 21% | 61 | 33% | 4 | 27% | 96 | 23% | 83 | 23% | 11 | 10% | 13 | 30% | 183 | 34% | 9 | 20% | 14 | 13% | 353 | 37% | 80 | 22% | 6 | 35% | 9 | 14% | 29 | 22% | 127 | 22% |  |
| Medical history |  |  |  |  |  |  |  |  |  |  |  |  |  |  |  |  |  |  |  |  |  |  |  |  |  |  |  |  |  |  |  |  |  |  | <0.001 |
| Healthy | 52 | 48% | 175 | 59% | 87 | 48% | 11 | 73% | 255 | 62% | 204 | 56% | 84 | 74% | 19 | 44% | 292 | 54% | 18 | 40% | 83 | 79% | 510 | 53% | 252 | 69% | 12 | 71% | 48 | 76% | 79 | 59% | 171 | 30% |  |
| Mild | 46 | 42% | 72 | 24% | 51 | 28% | 4 | 27% | 101 | 25% | 132 | 36% | 20 | 18% | 15 | 35% | 202 | 37% | 18 | 40% | 13 | 12% | 303 | 31% | 82 | 22% | 5 | 29% | 11 | 17% | 42 | 31% | 284 | 49% |  |
| Severe | 11 | 10% | 27 | 9% | 25 | 14% | 0 | 0% | 52 | 13% | 26 | 7% | 2 | 2% | 7 | 16% | 41 | 8% | 2 | 4% | 3 | 3% | 133 | 14% | 29 | 8% | 0 | 0% | 0 | 0% | 10 | 7% | 91 | 16% |  |
| Missing | 0 | 0% | 24 | 8% | 20 | 11% | 0 | 0% | 2 | 0% | 5 | 1% | 8 | 7% | 2 | 5% | 5 | 1% | 7 | 16% | 6 | 6% | 134 | 14% | 3 | 1% | 0 | 0% | 4 | 6% | 3 | 2% | 32 | 6% |  |
| Cause of injury |  |  |  |  |  |  |  |  |  |  |  |  |  |  |  |  |  |  |  |  |  |  |  |  |  |  |  |  |  |  |  |  |  |  | <0.001 |
| Road traffic accident | 9 | 8% | 111 | 37% | 66 | 36% | 6 | 40% | 171 | 42% | 110 | 30% | 49 | 43% | 14 | 33% | 270 | 50% | 16 | 36% | 13 | 12% | 358 | 37% | 141 | 39% | 9 | 53% | 23 | 37% | 43 | 32% | 210 | 36% |  |
| Fall | 76 | 70% | 126 | 42% | 88 | 48% | 6 | 40% | 177 | 43% | 177 | 48% | 52 | 46% | 20 | 47% | 192 | 36% | 21 | 47% | 28 | 27% | 502 | 52% | 153 | 42% | 3 | 18% | 25 | 40% | 55 | 41% | 254 | 44% |  |
| Violence | 3 | 3% | 11 | 4% | 5 | 3% | 0 | 0% | 20 | 5% | 26 | 7% | 5 | 4% | 6 | 14% | 15 | 3% | 5 | 11% | 32 | 30% | 27 | 3% | 27 | 7% | 2 | 12% | 8 | 13% | 6 | 4% | 46 | 8% |  |
| Self-harm | 0 | 0% | 4 | 1% | 1 | 1% | 0 | 0% | 11 | 3% | 2 | 1% | 3 | 3% | 0 | 0% | 10 | 2% | 0 | 0% | 0 | 0% | 6 | 1% | 1 | 0% | 0 | 0% | 2 | 3% | 2 | 1% | 6 | 1% |  |
| Other | 17 | 16% | 26 | 9% | 19 | 10% | 2 | 13% | 23 | 6% | 35 | 10% | 5 | 4% | 1 | 2% | 43 | 8% | 1 | 2% | 25 | 24% | 59 | 6% | 36 | 10% | 3 | 18% | 5 | 8% | 14 | 10% | 48 | 8% |  |
| Missing | 4 | 4% | 20 | 7% | 4 | 2% | 1 | 7% | 8 | 2% | 17 | 5% | 0 | 0% | 2 | 5% | 10 | 2% | 2 | 4% | 7 | 7% | 10 | 1% | 8 | 2% | 0 | 0% | 0 | 0% | 14 | 10% | 14 | 2% |  |
| TBI severity |  |  |  |  |  |  |  |  |  |  |  |  |  |  |  |  |  |  |  |  |  |  |  |  |  |  |  |  |  |  |  |  |  |  | <0.001 |
| Mild | 62 | 57% | 187 | 63% | 124 | 68% | 8 | 53% | 298 | 73% | 275 | 75% | 35 | 31% | 27 | 63% | 306 | 57% | 24 | 53% | 77 | 73% | 671 | 70% | 275 | 75% | 12 | 71% | 56 | 89% | 61 | 46% | 356 | 62% |  |
| Moderate | 13 | 12% | 23 | 8% | 13 | 7% | 2 | 13% | 18 | 4% | 33 | 9% | 12 | 11% | 3 | 7% | 79 | 15% | 5 | 11% | 4 | 4% | 83 | 9% | 25 | 7% | 1 | 6% | 1 | 2% | 18 | 13% | 38 | 7% |  |
| Severe | 34 | 31% | 62 | 21% | 38 | 21% | 5 | 33% | 91 | 22% | 43 | 12% | 59 | 52% | 5 | 12% | 149 | 28% | 15 | 33% | 4 | 4% | 176 | 18% | 57 | 16% | 1 | 6% | 6 | 10% | 45 | 34% | 172 | 30% |  |
| Missing | 0 | 0% | 26 | 9% | 8 | 4% | 0 | 0% | 3 | 1% | 16 | 4% | 8 | 7% | 8 | 19% | 6 | 1% | 1 | 2% | 20 | 19% | 32 | 3% | 9 | 2% | 3 | 18% | 0 | 0% | 10 | 7% | 12 | 2% |  |
| Brain AIS |  |  |  |  |  |  |  |  |  |  |  |  |  |  |  |  |  |  |  |  |  |  |  |  |  |  |  |  |  |  |  |  |  |  | <0.001 |
| Minor | 0 | 0% | 39 | 13% | 28 | 15% | 1 | 7% | 98 | 24% | 42 | 11% | 1 | 1% | 12 | 28% | 109 | 20% | 0 | 0% | 31 | 30% | 214 | 22% | 29 | 8% | 5 | 29% | 25 | 40% | 9 | 7% | 160 | 28% |  |
| Moderate | 0 | 0% | 22 | 7% | 24 | 13% | 1 | 7% | 70 | 17% | 115 | 31% | 1 | 1% | 2 | 5% | 39 | 7% | 0 | 0% | 15 | 14% | 75 | 8% | 77 | 21% | 1 | 6% | 13 | 21% | 11 | 8% | 37 | 6% |  |
| Serious | 0 | 0% | 68 | 23% | 58 | 32% | 2 | 13% | 108 | 26% | 97 | 26% | 4 | 4% | 9 | 21% | 99 | 18% | 22 | 49% | 48 | 46% | 372 | 39% | 127 | 35% | 9 | 53% | 18 | 29% | 24 | 18% | 118 | 20% |  |
| Severe | 79 | 72% | 67 | 22% | 34 | 19% | 3 | 20% | 80 | 20% | 54 | 15% | 30 | 26% | 10 | 23% | 106 | 20% | 10 | 22% | 3 | 3% | 116 | 12% | 60 | 16% | 2 | 12% | 2 | 3% | 16 | 12% | 43 | 7% |  |
| Critical | 29 | 27% | 47 | 16% | 34 | 19% | 6 | 40% | 50 | 12% | 58 | 16% | 76 | 67% | 10 | 23% | 171 | 32% | 13 | 29% | 8 | 8% | 145 | 15% | 65 | 18% | 0 | 0% | 4 | 6% | 70 | 52% | 214 | 37% |  |
| Unsurvivable | 1 | 1% | 2 | 1% | 5 | 3% | 0 | 0% | 4 | 1% | 1 | 0% | 2 | 2% | 0 | 0% | 15 | 3% | 0 | 0% | 0 | 0% | 40 | 4% | 8 | 2% | 0 | 0% | 1 | 2% | 1 | 1% | 6 | 1% |  |
| Missing | 0 | 0% | 53 | 18% | 0 | 0% | 2 | 13% | 0 | 0% | 0 | 0% | 0 | 0% | 0 | 0% | 1 | 0% | 0 | 0% | 0 | 0% | 0 | 0% | 0 | 0% | 0 | 0% | 0 | 0% | 3 | 2% | 0 | 0% |  |
| ISS |  |  |  |  |  |  |  |  |  |  |  |  |  |  |  |  |  |  |  |  |  |  |  |  |  |  |  |  |  |  |  |  |  |  | <0.001 |
| Minor (≤16) | 53 | 49% | 113 | 38% | 79 | 43% | 8 | 53% | 241 | 59% | 252 | 69% | 13 | 11% | 22 | 51% | 207 | 38% | 31 | 69% | 88 | 84% | 503 | 52% | 205 | 56% | 10 | 59% | 52 | 83% | 43 | 32% | 256 | 44% |  |
| Major (16-25) | 34 | 31% | 47 | 16% | 44 | 24% | 4 | 27% | 62 | 15% | 67 | 18% | 25 | 22% | 8 | 19% | 104 | 19% | 8 | 18% | 12 | 11% | 218 | 23% | 65 | 18% | 6 | 35% | 6 | 10% | 42 | 31% | 110 | 19% |  |
| Critically injured (>25) | 22 | 20% | 86 | 29% | 60 | 33% | 3 | 20% | 107 | 26% | 48 | 13% | 76 | 67% | 13 | 30% | 228 | 42% | 6 | 13% | 5 | 5% | 241 | 25% | 96 | 26% | 1 | 6% | 5 | 8% | 47 | 35% | 212 | 37% |  |
| Missing | 0 | 0% | 52 | 17% | 0 | 0% | 0 | 0% | 0 | 0% | 0 | 0% | 0 | 0% | 0 | 0% | 1 | 0% | 0 | 0% | 0 | 0% | 0 | 0% | 0 | 0% | 0 | 0% | 0 | 0% | 2 | 1% | 0 | 0% |  |
| Baseline pupillary reaction |  |  |  |  |  |  |  |  |  |  |  |  |  |  |  |  |  |  |  |  |  |  |  |  |  |  |  |  |  |  |  |  |  |  | <0.001 |
| Both reacting | 90 | 83% | 206 | 69% | 158 | 86% | 13 | 87% | 371 | 90% | 305 | 83% | 79 | 69% | 33 | 77% | 472 | 87% | 37 | 82% | 84 | 80% | 838 | 87% | 303 | 83% | 13 | 76% | 62 | 98% | 99 | 74% | 491 | 85% |  |
| One reacting | 1 | 1% | 20 | 7% | 9 | 5% | 1 | 7% | 16 | 4% | 10 | 3% | 4 | 4% | 0 | 0% | 13 | 2% | 4 | 9% | 1 | 1% | 35 | 4% | 13 | 4% | 0 | 0% | 0 | 0% | 7 | 5% | 28 | 5% |  |
| Both unreacting | 16 | 15% | 19 | 6% | 7 | 4% | 0 | 0% | 22 | 5% | 14 | 4% | 13 | 11% | 6 | 14% | 29 | 5% | 1 | 2% | 0 | 0% | 74 | 8% | 28 | 8% | 1 | 6% | 1 | 2% | 19 | 14% | 27 | 5% |  |
| Missing | 2 | 2% | 53 | 18% | 9 | 5% | 1 | 7% | 1 | 0% | 38 | 10% | 18 | 16% | 4 | 9% | 26 | 5% | 3 | 7% | 20 | 19% | 15 | 2% | 22 | 6% | 3 | 18% | 0 | 0% | 9 | 7% | 32 | 6% |  |
| CT abnormalities |  |  |  |  |  |  |  |  |  |  |  |  |  |  |  |  |  |  |  |  |  |  |  |  |  |  |  |  |  |  |  |  |  |  |  |
| Any intracranial abnormality | 99 | 91% | 175 | 59% | 92 | 50% | 3 | 20% | 256 | 62% | 188 | 51% | 101 | 89% | 19 | 44% | 344 | 64% | 3 | 7% | 17 | 16% | 492 | 51% | 185 | 51% | 6 | 35% | 29 | 46% | 98 | 73% | 281 | 49% | <0.001 |
| Cisternal compression | 19 | 17% | 34 | 11% | 26 | 14% | 1 | 7% | 45 | 11% | 40 | 11% | 33 | 29% | 6 | 14% | 96 | 18% | 0 | 0% | 4 | 4% | 164 | 17% | 25 | 7% | 0 | 0% | 5 | 8% | 42 | 31% | 87 | 15% | <0.001 |
| Midline shift | 13 | 12% | 22 | 7% | 15 | 8% | 1 | 7% | 25 | 6% | 35 | 10% | 26 | 23% | 4 | 9% | 70 | 13% | 0 | 0% | 4 | 4% | 126 | 13% | 21 | 6% | 2 | 12% | 4 | 6% | 29 | 22% | 58 | 10% | <0.001 |
| Subarachnoid hemorrhage | 84 | 77% | 134 | 45% | 72 | 39% | 2 | 13% | 192 | 47% | 128 | 35% | 86 | 75% | 15 | 35% | 274 | 51% | 1 | 2% | 9 | 9% | 359 | 37% | 122 | 33% | 3 | 18% | 24 | 38% | 80 | 60% | 208 | 36% | <0.001 |
| Epidural hematoma | 14 | 13% | 31 | 10% | 12 | 7% | 1 | 7% | 36 | 9% | 29 | 8% | 30 | 26% | 4 | 9% | 63 | 12% | 1 | 2% | 3 | 3% | 77 | 8% | 24 | 7% | 1 | 6% | 9 | 14% | 22 | 16% | 68 | 12% | <0.001 |
| Acute subdural heamtoma | 45 | 41% | 54 | 18% | 51 | 28% | 2 | 13% | 96 | 23% | 98 | 27% | 52 | 46% | 10 | 23% | 174 | 32% | 1 | 2% | 11 | 10% | 255 | 27% | 66 | 18% | 3 | 18% | 9 | 14% | 59 | 44% | 140 | 24% | <0.001 |
| Diffuse axonal injury | 17 | 16% | 39 | 13% | 14 | 8% | 1 | 7% | 41 | 10% | 22 | 6% | 25 | 22% | 3 | 7% | 50 | 9% | 0 | 0% | 1 | 1% | 74 | 8% | 61 | 17% | 0 | 0% | 2 | 3% | 21 | 16% | 72 | 12% | <0.001 |
| Contusion | 52 | 48% | 101 | 34% | 64 | 35% | 3 | 20% | 126 | 31% | 95 | 26% | 74 | 65% | 13 | 30% | 207 | 38% | 2 | 4% | 4 | 4% | 255 | 27% | 101 | 28% | 2 | 12% | 12 | 19% | 57 | 43% | 168 | 29% | <0.001 |
| Inhospital mortality |  |  |  |  |  |  |  |  |  |  |  |  |  |  |  |  |  |  |  |  |  |  |  |  |  |  |  |  |  |  |  |  |  |  | <0.001 |
| Alive | 95 | 87% | 234 | 79% | 113 | 62% | 12 | 80% | 261 | 64% | 275 | 75% | 102 | 89% | 26 | 60% | 407 | 75% | 43 | 96% | 78 | 74% | 719 | 75% | 277 | 76% | 14 | 82% | 38 | 60% | 108 | 81% | 414 | 72% |  |
| Dead | 14 | 13% | 16 | 5% | 16 | 9% | 2 | 13% | 20 | 5% | 2 | 1% | 12 | 11% | 6 | 14% | 48 | 9% | 2 | 4% | 1 | 1% | 101 | 10% | 19 | 5% | 0 | 0% | 6 | 10% | 6 | 4% | 39 | 7% |  |
| Missing | 0 | 0% | 48 | 16% | 54 | 30% | 1 | 7% | 129 | 31% | 90 | 25% | 0 | 0% | 11 | 26% | 85 | 16% | 0 | 0% | 26 | 25% | 142 | 15% | 70 | 19% | 3 | 18% | 19 | 30% | 20 | 15% | 125 | 22% |  |
| GOSE-6 months disability |  |  |  |  |  |  |  |  |  |  |  |  |  |  |  |  |  |  |  |  |  |  |  |  |  |  |  |  |  |  |  |  |  |  | <0.001 |
| 1 | 23 | 21% | 30 | 10% | 21 | 11% | 2 | 13% | 24 | 6% | 15 | 4% | 12 | 11% | 8 | 19% | 73 | 14% | 6 | 13% | 6 | 6% | 141 | 15% | 28 | 8% | 0 | 0% | 6 | 10% | 14 | 10% | 61 | 11% |  |
| 2_or_3 | 9 | 8% | 12 | 4% | 12 | 7% | 0 | 0% | 32 | 8% | 21 | 6% | 6 | 5% | 1 | 2% | 74 | 14% | 4 | 9% | 3 | 3% | 46 | 5% | 19 | 5% | 2 | 12% | 2 | 3% | 21 | 16% | 50 | 9% |  |
| 4 | 9 | 8% | 17 | 6% | 1 | 1% | 1 | 7% | 15 | 4% | 13 | 4% | 8 | 7% | 0 | 0% | 22 | 4% | 3 | 7% | 0 | 0% | 33 | 3% | 9 | 2% | 1 | 6% | 1 | 2% | 9 | 7% | 32 | 6% |  |
| 5 | 4 | 4% | 23 | 8% | 1 | 1% | 0 | 0% | 36 | 9% | 35 | 10% | 20 | 18% | 2 | 5% | 38 | 7% | 4 | 9% | 3 | 3% | 71 | 7% | 37 | 10% | 1 | 6% | 0 | 0% | 13 | 10% | 51 | 9% |  |
| 6 | 4 | 4% | 40 | 13% | 14 | 8% | 0 | 0% | 23 | 6% | 27 | 7% | 11 | 10% | 0 | 0% | 55 | 10% | 8 | 18% | 0 | 0% | 78 | 8% | 63 | 17% | 1 | 6% | 5 | 8% | 13 | 10% | 41 | 7% |  |
| 7 | 8 | 7% | 46 | 15% | 10 | 5% | 1 | 7% | 85 | 21% | 63 | 17% | 16 | 14% | 1 | 2% | 66 | 12% | 5 | 11% | 8 | 8% | 211 | 22% | 66 | 18% | 0 | 0% | 7 | 11% | 13 | 10% | 52 | 9% |  |
| 8 | 26 | 24% | 77 | 26% | 59 | 32% | 4 | 27% | 127 | 31% | 166 | 45% | 14 | 12% | 4 | 9% | 161 | 30% | 14 | 31% | 62 | 59% | 283 | 29% | 128 | 35% | 6 | 35% | 24 | 38% | 29 | 22% | 141 | 24% |  |
| Missing | 26 | 24% | 53 | 18% | 65 | 36% | 7 | 47% | 68 | 17% | 27 | 7% | 27 | 24% | 27 | 63% | 51 | 9% | 1 | 2% | 23 | 22% | 99 | 10% | 16 | 4% | 6 | 35% | 18 | 29% | 22 | 16% | 150 | 26% |  |

## Supplementary Methods

Example of calculation for ambulance costs of patient with TBI treated in Germany

Step 1: define unit price of ambulance transportation (Supplementary Table S1)

€741

Step 2:  calculate unit price for 2017 using general consumer price index (Supplementary Table S2)

€741* 0.983 (inflation 2018) * 0.986 (inflation 2017)= €718 

Step 3: calculate country specific unit price for Germany from Dutch reference price using GDP-PPP (Supplementary Table S3)

(€718 * 1.278772379) (PPP for GDP in US Dollar of The Netherlands) * 0.745 (PPP for GDP in National currency per US Dollar of Germany)= €684 

Step 4: calculate total costs of ambulance transportation by multiplying country specific unit price with number of healthcare units used (i.e. number times ambulance is used) 

€684 * 1

Total costs of using the ambulance service once for a patient with TBI treated in Germany is €684
